# Combination of the Novel RAF Dimer Inhibitor Brimarafenib With the MEK Inhibitor Mirdametinib Is Effective Against NRAS Mutant Melanoma

**DOI:** 10.1111/pcmr.70062

**Published:** 2025-11-06

**Authors:** Flavia L. Tellenbach, Luzia Seiler, Mark Johnson, Hubert Rehrauer, Prachi Schukla, Julia Martinez‐Gomez, Corinne I. Stoffel, Adeela Kamal, Reinhard Dummer, Mitchell P. Levesque, Ossia M. Eichhoff

**Affiliations:** ^1^ Department of Dermatology University Hospital of Zurich, University of Zurich Zurich Switzerland; ^2^ SpringWorks Therapeutics Stamford Connecticut USA; ^3^ Functional Genomics Center Zurich University of Zurich Zurich Switzerland

## Abstract

Metastatic melanoma, the most aggressive form of skin cancer, accounts for the majority of skin cancer‐related deaths. While targeted kinase inhibitors have improved outcomes for patients with BRAF‐mutated melanomas, their efficacy is often short‐lived, and effective treatments for other mutations, such as NRAS, remain scarce. To address this clinical need, we investigated the combination of the novel panRAF inhibitor, brimarafenib, and the MEK inhibitor, mirdametinib, both of which target the MAPK pathway downstream of NRAS. This study demonstrates the efficacy of this combination in NRAS‐mutated melanoma and is currently also investigated in a phase I/IIa clinical study. In vitro, the brimarafenib and mirdametinib combination exhibited synergistic effects, significantly inhibiting the growth of patient‐derived NRAS‐mutated melanoma cell lines. A colony formation assay showed that this combination prevented the emergence of drug‐resistant clones, suggesting a strong potential to reduce disease relapse. Transcriptional and proteomic analyses revealed that the observed growth inhibition was due to modulation of MAPK signaling and induction of apoptosis. In vivo studies further validated these findings, showing that the combination treatment inhibited tumor growth and significantly prolonged survival in mouse models bearing patient‐derived NRAS‐mutated melanoma tumors. Given the tolerability of this combination in vivo, our results suggest that brimarafenib and mirdametinib represent a promising therapeutic strategy for patients with NRAS‐mutated melanomas and potentially other RAS‐mutated solid tumors.

## Graphical abstract

(A) NRAS mutations in melanoma lock the NRAS protein in its active state, driving constitutive activation of the MAPK pathway and promoting cell proliferation. Activation of RAF proteins by RAS mutations involves not only BRAF but also CRAF. (B) Inhibition of the downstream kinase MEK by mirdametinib reduces signaling to the ERK kinase but also diminishes negative feedback mechanisms that regulate pathway activation, leading to the accumulation of phosphorylated MEK protein. (C) In combination with the pan‐RAF inhibitor brimarafenib, which prevents heterodimerization with CRAF and thereby inhibits RAF activation, unphosphorylated MEK binds to CRAF, resulting in pathway shutdown and apoptosis. (Created in BioRender. Restivo, G. (2025) https://BioRender.com/1sf64h9).

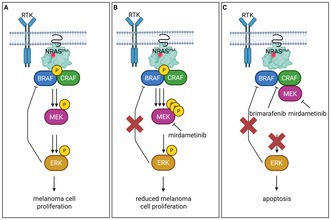

**Summary**

- The novel RAF dimer inhibitor brimarafenib combined with the MEK inhibitor mirdametinib is effective against NRAS‐mutated melanoma.
- The combination therapy shows significant synergy in inhibiting tumor growth in both in vitro and in vivo models.
- This therapeutic approach effectively suppresses residual tumor cell survival in long‐term colony formation assay
- The combination leads to sustained inhibition of the MAPK pathway.
- The results highlight the potential of this combination therapy to meet the need for effective treatments in patients with NRAS mutations or other RAS‐mutated tumors.

## 1 Introduction

The mitogen activated protein kinase (MAPK) pathway plays a critical role in cellular growth, gene expression, and survival. In cancer, this pathway represents a key therapeutic area of interest (Samatar and Poulikakos 2014). Mutations within the MAPK pathway occur across various cancer types, with RAS mutations present in about one‐third of all cancers (Drosten and Barbacid 2020). In melanoma, over 80% of patients have MAPK pathway mutations, emphasizing its importance in melanoma development (Leonardi et al. 2018). The BRAF protein, a MAPK pathway member, is the most commonly mutated in melanoma, with the V600E mutation occurring in about 50% of cases. (Long et al. 2011). Hyper‐activated BRAF protein leads to activation of downstream MAPK pathway signaling, promoting cancer growth (Wellbrock et al. 2004). Another member of the MAPK pathway, the GTPase NRAS, is observed to be mutated in about 30% of all melanoma cases, with the most common mutations in either exon 1 (G12 or G13) or exon 2 (Q61). Mutations in NRAS leads to hyper‐activated kinase activity that functions independently of upstream receptor tyrosine kinase (RTK) activation (Hayward et al. 2017; Vu and Aplin 2016).

Targeted therapies have been developed for BRAF‐mutated melanoma, notably vemurafenib, which showed significant improvement in response rates and overall survival compared to traditional chemotherapy and became the first targeted standard of care for BRAF mutated melanoma (McArthur et al. 2014).

However, resistance often develops due to mechanisms like BRAF amplification, RAF dimerization, or additional mutations in MEK and NRAS. To address this, a combination of BRAF and MEK inhibitors has become the standard treatment, providing better outcomes by delaying resistance and reducing secondary malignancies in non BRAF‐mutated cells (Shi et al. 2014; Raaijmakers et al. 2016; Rebocho and Marais 2013) (Larkin et al. 2014; Long et al. 2014).

Patients with NRAS‐mutated melanoma generally have poorer outcomes, with a higher likelihood of brain metastases (Thumar et al. 2014). Unlike BRAF, NRAS lacks a potent, specific inhibitor, making immunotherapy the primary treatment. Although immunotherapy shows promise, with a 2‐year overall survival rate of around 54%, many patients do not respond, leaving limited treatment options. (Zaremba et al. 2023). The MEK inhibitor binimetinib has shown some efficacy, with partial responses in 20% of patients and a progression‐free survival of 3.7 months (Dummer et al. 2017). However, RAS‐mutated cells are generally less sensitive to MEK inhibition due to alternative activation pathways involving CRAF (Lito et al. 2014). Combining MEK inhibitors with pan‐RAF inhibitors may enhance effectiveness against NRAS mutations.

Mirdametinib, a selective MEK1/2 inhibitor, has shown promising results in clinical trials. In a phase I study involving advanced solid tumors, mirdametinib significantly reduced pERK and Ki67 levels in melanoma patients, including those with NRAS mutations. The drug demonstrated partial responses in melanoma patients, suggesting its potential utility in treating NRAS‐mutated cancers. Further studies are evaluating the combination of mirdametinib with the RAF inhibitor lifirafenib in patients with MAPK‐activating mutations, showing encouraging results across various cancer types (LoRusso et al. 2010). Clinical results from this Phase 1b study support the evaluation of mirdametinib in combination with RAF inhibitors (Solomon et al. 2023).

Brimarafenib (BGB‐3245) is a next generation RAF inhibitor that has exhibited potent effects against different RAF isoforms, including BRAF Class I/II/III mutations, BRAF fusions, and heterodimerization with CRAF in preclinical models. In a Phase 1a/1b clinical trial, monotherapy treatment with brimarafenib resulted in a disease control rate (DCR) of 48% and an overall response rate (ORR) of 18%, with notable responses in patients with NRAS‐mutated melanoma. (Schram et al. 2023).

In this present study we demonstrate that dual inhibition of the MAPK pathway with brimarafenib and mirdametinib is effective against NRAS‐mutated metastatic melanoma in vitro. The combination therapy provided superior anti‐tumor efficacy compared to monotherapy alone in in vivo models. These findings support further clinical investigation of this combination in NRAS‐mutated melanoma patients, including those resistant to other MEK inhibitors.

## 2 Materials and Methods

### 2.1 Cell Culture and Reagents

Tissue biopsies were collected with written informed consent, and melanoma cells were isolated as described previously (Raaijmakers et al. 2015). Primary melanoma cell cultures were maintained in RPMI 1640 (Sigma‐Aldrich; R0883) with supplements: 5 nM L‐glutamine (Gibco, Thermo Scientific; 25030‐024), 1 mM sodium pyruvate (Sigma‐Aldrich; S8636), 10% heat‐inactivated fetal bovine serum (Biowest; S181H) and 1% Pen Strep (Gibco, Thermo Scientific; 15140‐122) at 37°C with 5% CO_2_.

Brimarafenib and mirdametinib was provided by SpringWorks Therapeutics Inc. (Stamford, CT). Binimetinib was purchased from Selleckchem (S7007). All compounds were dissolved in 100% DMSO to a stock concentration of 10 mM. Vehicle‐treated controls contained a final concentration of 0.1% DMSO for single‐agent treatments and 0.2% DMSO for combination treatments, respectively.

### 2.2 IC_50_ and Drug Synergy Analysis

Melanoma cells were plated in 96‐well plates (1.5 × 10^3^ cells/well) and treated with brimarafenib or mirdametinib for 72 h. Cell viability was assessed using Resazurin sodium salt (ACROS Organics; 41890‐0010) and fluorescence was measured at 530–560 nm (excitation) and 590 nm (emission) (Tecan, Infinite M200 Pro). IC50 values were calculated using GraphPad Prism, and drug synergy was analyzed with the SynergyFinder algorithm (https://synergyfinder.org).

### 2.3 Apoptosis Assay

Melanoma cells were seeded in 6‐well plates (1 × 10^5^ cells/well) and treated with brimarafenib, mirdametinib, or their combination for 48 h. Cells were stained with propidium iodide and the FITC Annexin V Apoptosis Detection Kit (BioLegend; 640914) according to the manufacturer's protocol. Samples were acquired using a LSR II Fortessa (BD Bioscience) and data were processed with FlowJo software (BD Bioscience).

### 2.4 Western Blotting

Cells were treated for 24 or 72 h, and proteins were extracted using RIPA lysis buffer. Protein concentrations were determined (DC Protein Assay, BioRad; 500‐0114). About 20 μg of total protein of each sample was separated via SDS‐PAGE and transferred to nitrocellulose membranes. Membranes were blocked, probed with primary antibodies overnight at 4°C, incubated with HRP‐conjugated secondary antibodies, and imaged for chemiluminescence (LI‐COR Odyssey Fc). Antibodies used included: phospho‐ERK/2 (Cell Signaling Technology (CST); 9101; 1:1000), ERK (CST; 9102; 1:1000), phospho‐MEK1/2 (CST; 9154; 1:1000), MEK (CST; 8727; 1:1000), SPRY1 (CST; 12,993; 1:500), DUSP6 (abcam; ab76310; 1:1000), CCND1 (CST; 55506; 1:500), PARP (CST; 9542; 1:1000), and HSP90 (CST; 4877; 1:2000).

### 2.5 Immunoprecipitation

Melanoma cells were treated for 24 h, lysed, and protein concentrations were measured (DC Protein Assay, BioRad; 500‐0114). About 0.8–1.0 mg of protein was incubated overnight with antibodies against MEK1 (CST, #2352). Immunoprecipitation was performed using protein A/G magnetic beads (Thermo Fischer Scientific, 78609) pre‐washed with blocking buffer (PBS, 1% bovine serum albumin (BSA) and 0.1% Tween‐20). Samples were eluted, heated at 80°C, and analyzed by Western Blotting using antibodies against MEK1/2 (CST, #8727), phospho‐MEK1/2 (CST, #9154) and RAF‐1 (CRAF, CST, #12552S).

### 2.6 RNA Sequencing and Data Processing

High‐quality RNA was extracted using the “high pure RNA isolation kit” (Roche, Cat#11828665001). Total RNA was subsequently submitted to the Functional Genomic Center Zurich for further processing and data analysis (differential expression and pathway enrichment) using R language and clusterProfiler package (v4.0.5) (Team, R.C 2023; Yu et al. 2012). The analysis was conducted using the enrichGO function and treatment exclusive pathways were extracted using Venny 2.1 (Oliveros 2007–2015). GO_Terms were visualized using the R‐package ggplot2 (Wickham 2016).

### 2.7 Colony Formation Assay

Melanoma cells were plated in 12‐well plates (0.5–1.0 × 10^3^ cells/well) and treated with brimarafenib, mirdametinib, or both. Drug concentrations were based on clinical trial data. Colonies were grown for 2 weeks, stained with colony fixation‐staining solution (formaldehyde 6%, crystal violet 0.5%), and scanned at 600 dpi (Epson Perfection V850 Pro) (Brix et al. 2021). Colony formation was quantified using ImageJ software.plug in Colony Area (Guzman et al. 2014).

### 2.8 In Vivo Efficacy Study

5 × 10^5^ of M130227 cells or 1 × 10^4^ of M161227 cells were injected with Matrigel (Corning, Cat# CLS356234, dilution 1:1) into the flanks of BALB/c nude mice (Charles River, Germany). Mice were randomized to treatment groups (*n* = 8) when tumors reached 0.1 cm^3^ in size. Tumor‐bearing animals were treated with brimarafenib (2 mg/kg PO, BID [for M130227]) or (1.5 mg/kg PO, BID [for M161227]), mirdametinib (0.5 mg/kg PO, BID [for M130227] or 0.3 mg/kg PO, BID [for M161227]), or the combination. Tumor growth was monitored two times per week. Mice were euthanized when tumors reached 1.5 cm^3^, or when termination criteria were fulfilled, whichever came first. For pharmacodynamic assessment, tumors were grown until they reached 0.5 cm^3^ and two mice per treatment group were given three doses of the assigned drug combination over approximately 36 h. One hour after the last treatment mice were euthanized and tumors were excised, fixed in formalin, and paraffin‐embedded for downstream immunohistochemical analysis. All animal experiments were performed by EPO (Berlin, Germany).

### 2.9 Immunohistochemistry

Formalin‐fixed paraffin‐embedded tumor slides were stained for S100 (Leica, Clone NCL‐l‐S100p; dilution 1:600) and pERK (CST; 4376; 1:1000) antibodies and counterstained with hematoxylin (Leica, Bond Polymer Refine Red Detection Kit; DS9390), and analyzed using Aperio ImageScope software (distributed by Leica Biosystems). Immunohistochemistry was scored using the QuPath software (Bankhead et al. 2017).

### 2.10 Statistical Analysis

All experiments were performed with at least three technical replicates. Unless stated otherwise, a Student's *t*‐test or two‐way ANOVA was used and data were analyzed with R programing language or with GraphPad Prism software.

### 2.11 Study Approval

Melanoma biopsies and clinical data were collected by the University Hospital Zurich Melanoma Biobank with informed consent and IRB approval (BASEC‐2017‐0494). All animal procedures were approved by the Institutional Animal Care and Use Committee (IACUC).

## 3 Results

### 3.1 NRAS Mutated Melanoma Cell Proliferation Is Inhibited by Combination of the RAF Dimer Inhibitor, Brimarafenib, and the MEKi, Mirdametinib

**FIGURE 1:**
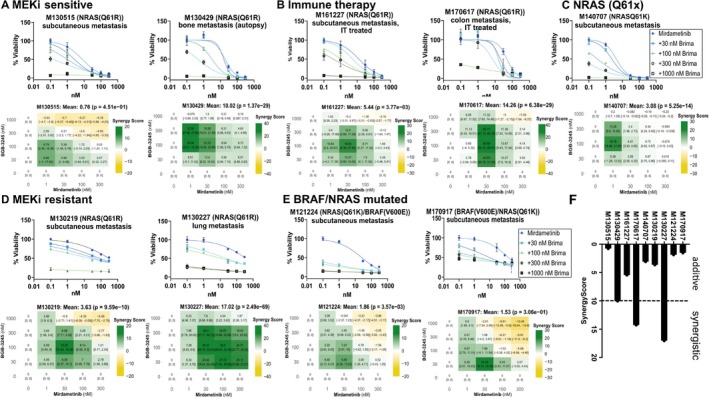
Brimarafenib in combination with mirdametinib effectively inhibits proliferation of NRAS‐mutated melanoma. (A–E) Patient‐derived melanoma cell lines were treated with brimarafenib and mirdametinib in a dose–response matrix. The upper panel shows dose–response curves for cells treated with mirdametinib and increasing concentrations of brimarafenib. The lower panel shows heatmaps including the mean ZIP synergy score and the corresponding *p* value. Heatmaps were generated using the SynergyFinder algorithm (https://synergyfinder.org). (F) Summary of synergy scores for each cell line. All experiments were performed in technical triplicates, and data were combined from two independent experiments.

A panel of patient‐derived melanoma cell lines with confirmed NRAS mutations were collected to study the combination effect of the RAF dimer inhibitor, brimarafenib, with the MEKi, mirdametinib. Patient characteristics, including oncogenic aberrations analyzed by MelArray next‐generation sequencing, are summarized in Table 1 (Hilbers et al. 2021; Freiberger et al. 2019). The melanoma cell lines, derived from patients harboring NRAS Q61R mutations (*n* = 6), showed varying responses to mirdametinib, reflecting their clinical history (M130219, M130227, M130429, M130515, M161227, M170617) (Figure 1, Figure S1). Three cell lines are derived from patients with NRAS Q61R mutations (M130219, M130227, M130429), who participated in the NEMO study and received binimetinib (Dummer et al. 2017). However, the biopsies from which M130219 and M130429 were created are derived from the same patient. Of note, the cell line M130219 was collected before start of the study and showed intrinsic resistance to the MEKi binimetinib during in vitro proliferation assays (Figure S1, (Eichhoff et al. 2023)). By contrast, the biopsy for M130429 was collected at autopsy and this cell line has shown sensitivity to MEKi. These intra‐patient derived cell lines represent a case study for cancer cell heterogeneity and phenotype‐switching, reported to be essential for melanoma progression and resistance to MAPK inhibitors (Karras et al. 2022; Rambow et al. 2018). The biopsy from M130227 was taken during the NEMO trial after the patient progressed on binimetinib treatment and, consequently, is less sensitive to MEKi Figure S1, (Eichhoff et al. 2023). The cell line M130515 was established from a cutaneous metastasis of a treatment naïve patient. The M161227 and M170617 cell lines were established from NRAS Q61R patients who received immunotherapy. The M161227 cell line was derived from a patient before receiving pembrolizumab and epacadostat. This patient showed progressive disease in the first 3 months of treatment, suggesting an intrinsic resistance to ITs. The M170617 cell line was established from a patient biopsy taken at the end of therapy after experiencing disease progression on nivolumab. M140707 (NRAS Q61K) was collected from a patient that presented with a new metastasis 3 years after responding to ipilimumab. Lastly, the M170917 and M121224 cell lines were obtained from patients harboring BRAF V600E mutated tumors that gained an NRAS Q61K mutation and progressed while undergoing encorafenib treatment. Melanoma tumors that harbor both BRAF and NRAS mutations have been shown to be resistant to BRAF inhibitor (BRAFi) monotherapy (Raaijmakers et al. 2016).

**TABLE 1:** Clinical characteristics of the nine patient‐derived melanoma cell lines. Mutational data was obtained using a custom‐next‐generation gene‐sequencing panel (MelArray, (Hilbers et al. 2021)).

	Cell culture	Gender	Age at biopsy	Oncogenic mutation	Additional oncogenic SNV	Additional oncogenic CNV	Biopsy location	Melanoma subtype	Last treatment before biopsy	MAPKi experience	Response to treatment	Time of biopsy
A primary melanoma cell line derived from patients with NRAS(Q61R) mutations												
MEKi resistant	M130219	m	47	NRAS(Q61R)	ARID1B(P1474S)	CDKN2A/B, MTAP (p16) deletion	Subcutaneous (chest)	Cutaneous (nodular)	Binimetinib	Yes	Resistant	Before the start of NEMO trial
	M130227	f	84	NRAS(Q61R)	BCLAF1(R429Q); (R111H)	NRAS amplification, MTAP (p16) deletion	Lung (pleura)	Cutaneous	Binimetinib	Yes	Resistant	During the NEMO trial
MEKi sensitive	M130429	m	48	NRAS(Q61R)	ARID1B(P1474S)	CDKN2A/MTAP (p16) deletion	Bone (autopsy)	Cutaneous (nodular)	Binimetinib	Yes	Resistant	After the NEMO trial (autopsy material)
	M130515	m	57	NRAS(Q61R)	GNAQ(T96S), GNAQ(Y101Term)	CDKN2A/B, GNAQ deletion	Cutaneous/subcutaneous (axilla)	Cutaneous (SSM)	Untreated	No	NA	From surgery material (removal of metastasis)

	Cell culture	Gender	Age at biopsy	Oncogenic mutation	Additional oncogenic SNV	Additional oncogenic CNV	Biopsy location	Melanoma subtype	Last treatment before biopsy	MAPKi experience	Response to treatment	Time of biopsy
B Primary melanoma cell lines derived from patients with NRAS(Q61R) mutations which progressed under IT												
IT	M170617	f	64	NRAS (Q61R)	p53(R196Term)	MYC amplification	Colon	Unclear	Nivolumab	No	Resistant	After progression to it
	M161227	m	64	NRAS (Q61R)	RET(R982C)	None	Subcutaneous (scapula)	Cutaneous	Pembrolizumab+ epacadostat	No	Resistant	Before the start of it

	Cell culture	Gender	Age at biopsy	Oncogenic mutation	Additional oncogenic SNV	Additional oncogenic CNV	Biopsy location	Melanoma subtype	Last treatment before biopsy	MAPKi experience	Response to treatment	Time of biopsy
C Primary melanoma cell line derived from patient with NRAS(Q61x)												
BRAF/NRAS	M121224	m	37	BRAF(V600E)	NRAS(Q61K)	CDKN2A/B, MTAP (p16), PTEN deletion	Subcutaneous (chest)	Cutaneous	Encorafenib	Yes	Resistant	At autopsy
	M170917	f	33	BRAF(V600E)	NRAS(Q61R)	CDKN2A/B, MTAP (p16) deletion	Subcuteanous (thorax)	Cutaneous	Encorafenib	Yes	Resistant	After progression from mapki

	Cell culture	Gender	Age at biopsy	Oncogenic mutation	Additional oncogenic SNV	Additional oncogenic CNV	Biopsy location	Melanoma subtype	Last treatment before biopsy	MAPKi experienced	Response to treatment	Time of biopsy
D Primary melanoma cell lines derived from patients originally diagnosed with BRAF(V600E) mutations but experienced MAPK resistance by NRAS(Q61x) mutations												
NRAS(Q61x)	M140707	f	75	NRAS(Q61K)	None	CDKN2A/B, MTAP (p16) deletion	Subcutanous (leg)	Cutaneous (nodular)	Ipilimumab	No	Sensitive	3 years after response to IT from new metastasis

Patient‐derived melanoma cell lines were evaluated after treatment with brimarafenib and mirdametinib using the dose–response matrix design and drug combination effects were analyzed using the SynergyFinder algorithm (Ianevski et al. 2020). As expected, cell lines deriving from patients whose tumors were resistant or sensitive to binimetinib showed corresponding IC_50_ value trends in response to mirdametinib treatment in vitro. Furthermore, varying levels of response to brimarafenib monotherapy treatment was seen in the cohort of NRAS mutated melanoma cell lines with IC_50_ values ranging from 34 to 670 nM (Figure S1A,B). Of note, the M121224 cell line, which is derived from a patient harboring both a BRAF and NRAS mutation, had the lowest IC_50_ value in response to brimarafenib alone. When brimarafenib was combined with mirdametinib, accumulating effect was observed across all patient‐derived melanoma cell lines, irrespective of NRAS mutational status and known resistance to MEKi. Synergistic scores were observed in cell lines M130227, M130429, and M170617, all of which harbor an NRAS Q61R mutation, while the remaining cell lines achieved varying scores consistent with additive synergy. Interestingly, the highest synergistic score observed was in the M130227 cell line, which was derived from a patient whose disease progressed while on binimetinib. (Figure 1A–F, Figure S1B). In summary, these data suggest a positive correlation in the response to combined mirdametinib and brimarafenib treatment in vitro, as measured by synergy proliferation assays.

### 3.2 Brimarafenib and Mirdametinib Combination Therapy Prevent the Outgrowth of Cells That Can Give Rise to Minimal Residual Disease and Cancer Relapse

**FIGURE 2:**
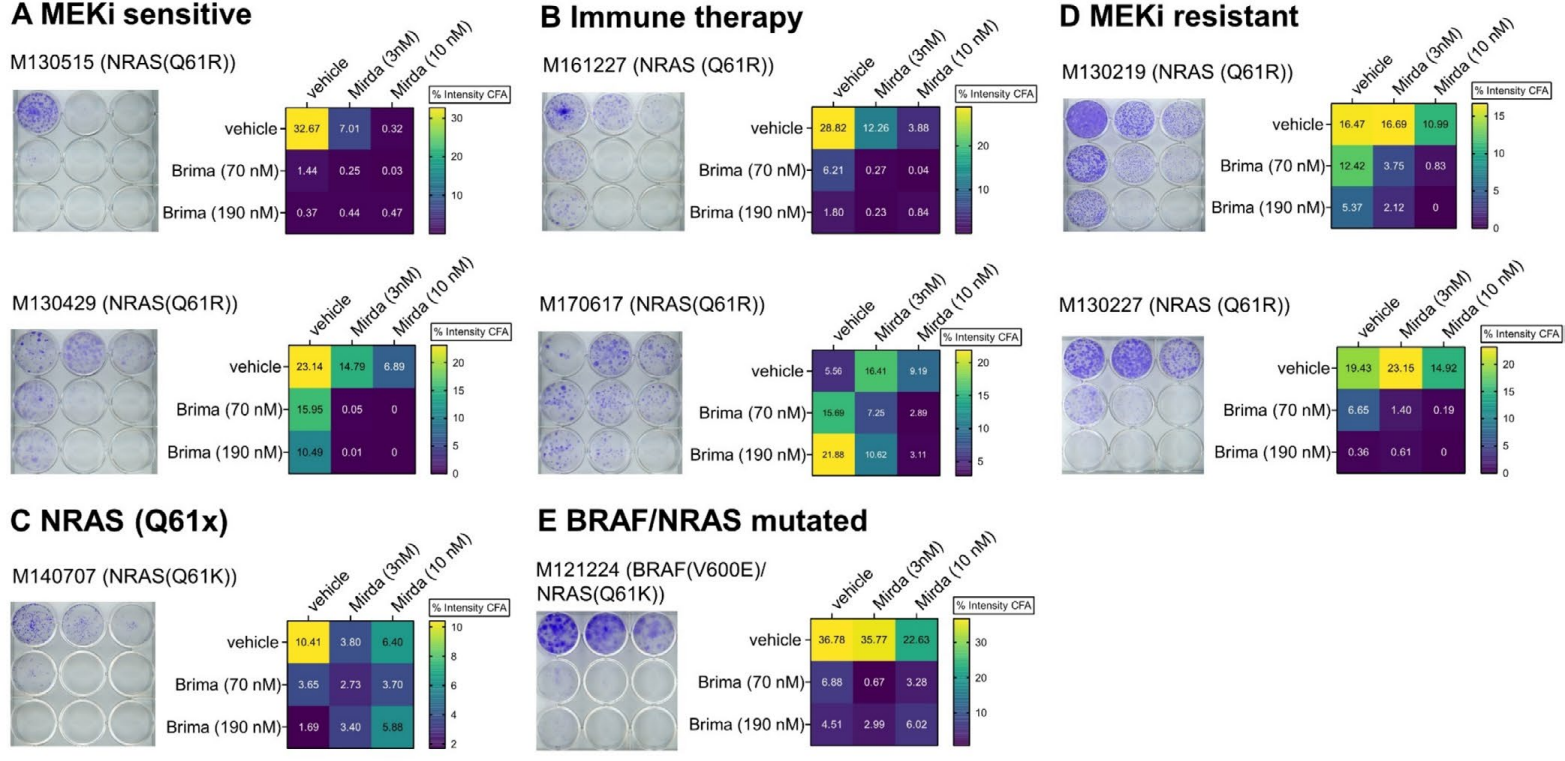
Brimarafenib and mirdametinib combination treatment inhibits colony formation. Patient‐derived melanoma cell lines were treated with 70 or 190 nM brimarafenib, 3 or 10 nM mirdametinib, or their combination and incubated for 2 weeks. Colony formation was assessed by crystal violet staining. Staining intensity was measured using a high‐density plate scanner, and images were analyzed using the *Colony Formation Analysis* plug‐in for ImageJ (Brix et al. 2021). Quantification results are presented as a heatmap generated in GraphPad Prism. Data were obtained from technical triplicates and two independent experiments. A representative plate is shown for each cell line.

Clonogenic assays, or colony formation assays (CFA), use patient derived melanoma cells, which were treated with clinically relevant doses of brimarafenib and mirdametinib, to evaluate whether combination treatment can eliminate melanoma cells with colony formation potential and self‐renewal capacity. While proliferation assays measure cell growth rate over a short period—primarily reflecting metabolic activity and cell division—CFAs are better suited to assess the long‐term effects of targeted therapies on cell viability and the potential for tumor regrowth (Franken et al. 2006). Significant reductions in colony numbers were observed across eight tested cell lines in the combination treatment conditions after 2 weeks, highlighting the effectiveness of brimarafenib and mirdametinib co‐treatment in this assay system, regardless of the origin of the cell line. (Figure 2, Figure S2). The only exception was cell line M170917, which did not form colonies under any condition in this assay. More specifically, outgrowth of colonies was seen following treatment with mirdametinib or brimarafenib alone in cell lines M130219 and M130227, both derived from patients with known resistance to the MEKi binimetinib (Figure 2E). This outcome is consistent with the clinical background of these cell lines. Additionally, cell lines derived from patients with immune therapy (IT) resistance (M161227, M170617, and M140707; Figure 2B,C) also showed outgrowth of single‐cell colonies under treatment with either mirdametinib or binimetinib alone, suggesting potential cross‐resistance between these treatments. Although the two MEKi‐sensitive cell lines, M130515 and M130429, display low IC_50_ values for mirdametinib in vitro (Figure S1B), in this clonogenic assay, M130429 formed colonies under both mirdametinib and brimarafenib treatment. M130429 was derived from a patient undergoing binimetinib treatment during the NEMO trial and was resistant to MEK inhibition, suggesting that drug‐resistant clones were already present in the biopsy. In contrast, M130515 is the only cell line derived from a treatment‐naïve patient and was also the only line that showed sensitivity to both compounds when used as monotherapy (Figure 2A).

**FIGURE 3:**
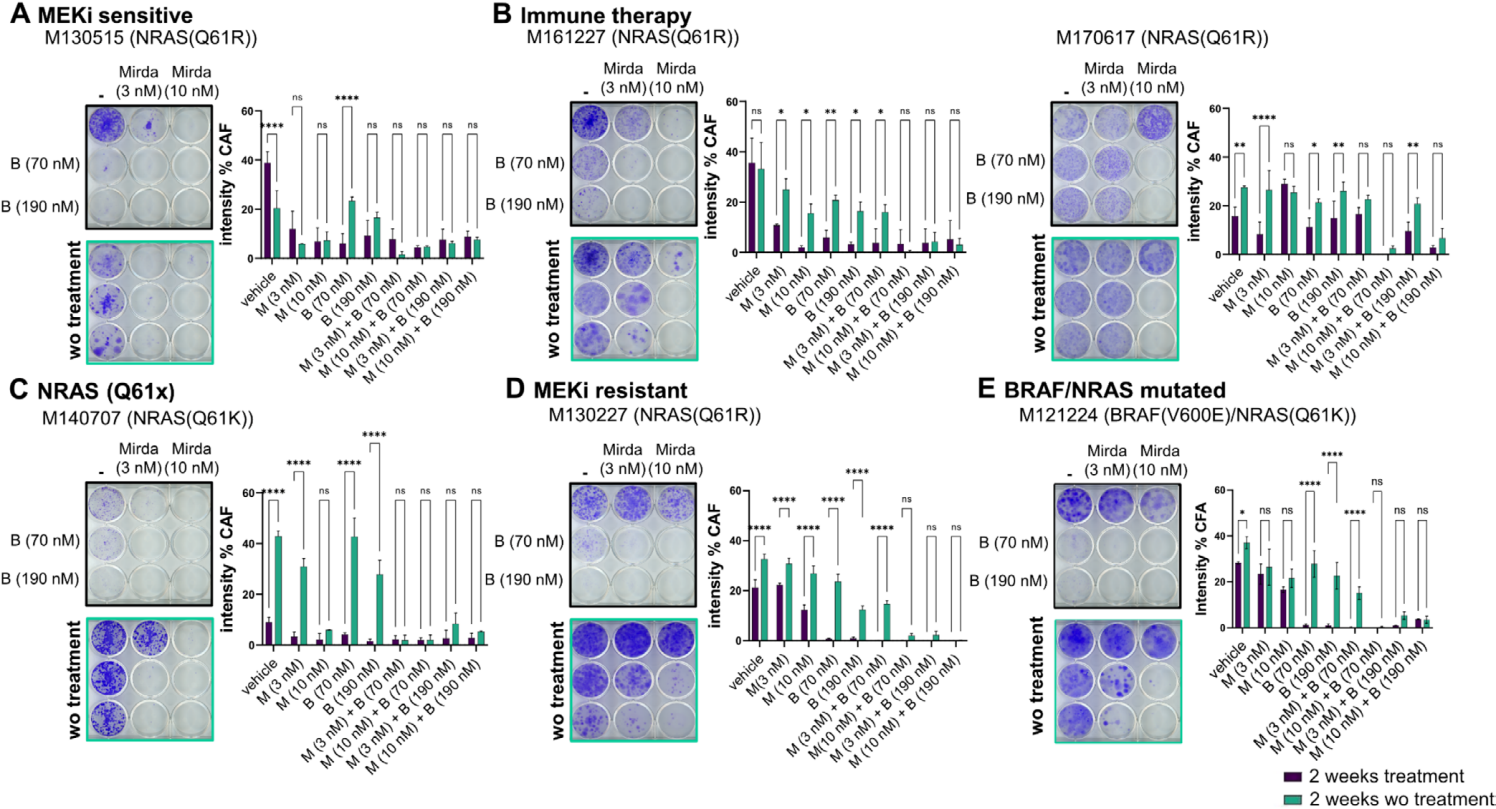
Brimarafenib and mirdametinib combination treatment prevents colony re‐growth after treatment removal. Patient‐derived melanoma cell lines were treated with 70 or 190 nM brimarafenib, 3 or 10 nM mirdametinib, or the combination for 2 weeks. Following treatment, cell lines were grown without treatment for two additional weeks. Crystal violet staining intensity was determined using a high‐density plate scanner and pictures were analyzed by the colony formation analysis plug‐in for ImageJ (Brix et al. 2021) and output data were plotted using GraphPad Prism software. Representative plates are shown for each cell line. Data was obtained from technical triplicates and two independent experiments. (p* < 0.05, p** < 0.01, p*** < 0.001, p**** < 0.0001, two‐way ANOVA).

Clones that survive treatment give rise to minimal residual disease (MRD) and spur the onset of disease relapse. This phenomenon has been described for many cancers including melanoma (Rambow et al. 2018; Ma et al. 2023). To evaluate this, patient‐derived melanoma cell lines were treated with brimarafenib, mirdametinib, or the combination for 2 weeks. After treatment removal, the cells were cultured for an additional 2 weeks to assess colony re‐growth (Figure 3). Significant re‐growth of melanoma colonies was seen in most cell lines when treated with brimarafenib or mirdametinib alone but not when treated with the combination. One exception was the MEKi sensitive cell line M130515, which is derived from a treatment naïve patient, and did not form colonies after removal of mirdametinib monotherapy. Overall, the combination of brimarafenib and mirdametinib significantly reduced the emergence of colonies potentially capable of driving MRD across all tested cell lines, underscoring the therapeutic advantage of this combination in a model of melanoma relapse.

### 3.3 RNA Sequencing Analysis Revealed Downregulation of MAPK Target Genes, Induction of Apoptosis, and Inhibition of DNA Synthesis After Brimarafenib and Mirdametinib Treatment

**FIGURE 4:**
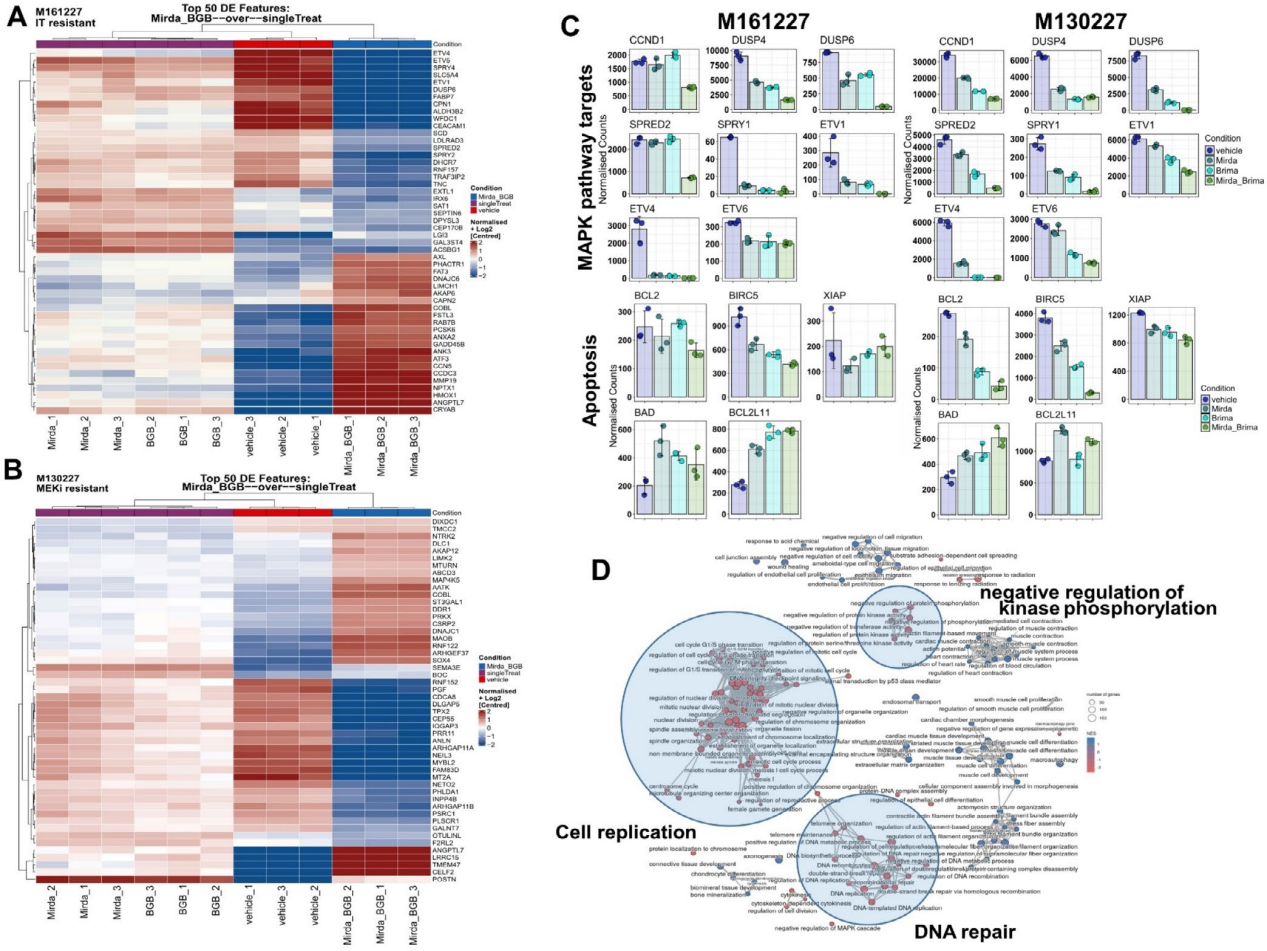
Combination treatment with brimarafenib and mirdametinib inhibits MAPK pathway activation and induces apoptotic pathways. Melanoma cell lines M161227 and M130227 were treated with 10 nM mirdametinib (Mirda), 190 nM brimarafenib (BGB), or the combination (Mirda_BGB) for 48 h before RNA isolation and sequencing. (A) Heatmap of log_2_‐transformed RNA transcript counts from M161227 (derived from a patient resistant to pembrolizumab and epacadostat). (B) Heatmap of log_2_‐transformed RNA transcript counts from M130227 (derived from a patient resistant to binimetinib). In both panels, the top 50 differentially expressed genes across all conditions are shown (FDR < 0.05). (C) Normalized counts of genes involved in MAPK signaling and apoptosis pathways. Differential expression analysis was performed comparing the combination treatment to vehicle control (FDR < 0.05; see Tables S1–, S3). (D) Network analysis of Gene Ontology (GO) Biological Processes from combined data, comparing combination‐treated to vehicle‐treated samples. Key pathways affected include DNA repair, cell replication, and negative regulation of kinase phosphorylation.

To explore the molecular mechanisms behind the observed synergistic effects, RNA sequencing was performed on two cell lines treated with brimarafenib and mirdametinib. (DEseq data available in Tables [Link], [Link], [Link]). One cell line used here is M161227, which was derived from a patient before receiving pembrolizumab and epacadostat but rapidly progressed under treatment. The other cell line is M130227, which was established from a patient's metastasis during binimetinib treatment showing progressive disease. We performed a differential expression analysis, which was then used to generate a heatmap of the top 50 differentially expressed genes. (adj. *p*‐value < 0.05; Figure 4A,B). We observed in both cell lines that, while single‐agent treatment with mirdametinib or brimarafenib altered gene expression to some extent but still clustered with the vehicle samples, only the combination of panRAF and MEK inhibition shifted gene expression in the opposite direction. Additionally, M161227 shows a strong enrichment of MAPK‐activated genes among the top 50 differentially expressed transcripts, which are upregulated in the vehicle samples (e.g., ETV4/5, SPRY4, DUSP6) (Dry et al. 2010; Joseph et al. 2010). The expression of these MAPK‐activated genes were significantly downregulated with the combination of brimarafenib and mirdametinib. In contrast, the vehicle samples of cell line M130227 predominantly upregulate factors that indirectly influence MAPK pathway activity through growth factor signaling and cell cycle progression, thereby promoting melanoma aggressiveness (e.g., PGF, CDCA8, DLGAP5, TPX2) (Li et al. 2023; Garrido and Vernos 2016; Ci et al. 2019; Pagani et al. 2016). Here, too, the combination treatment clusters opposite to the single drug treatments, suggesting that these factors are positively regulated to some extent by MAPK signaling. To focus on MAPK pathway regulation and the effects on key downstream factors, we highlight direct MAPK‐activated pathway target genes. We observed a heterogeneous response to brimarafenib or mirdametinib alone on MAPK target genes, such as CCND1 (Cyclin D1) and DUSP6. Only the combination treatment led to a significant downregulation of MAPK pathway target genes (adj. *p*‐value < 0.05, Figure 4B,C), suggesting that combination therapy is required to effectively suppress MAPK signaling. Furthermore, while interrogating modulations in apoptotic pathway genes, the anti‐apoptotic factors *BCLS*, *BIRC5*, and *XIAP* were all significantly downregulated with the combination treatment, while the pro‐apoptotic factors *BAD* and *BCL2L11* were significantly upregulated. Mechanistically, this highlights the cytotoxic effects of combination treatment as detected by transcriptional analysis (Hussein et al. 2003).

Finally, a two‐group network analysis of gene ontology terms (GO) of biological processes (BP) comparing vehicle‐treated control to combination treatment samples from both cell lines was performed to interrogate pathway modifications (Figure 4D). Network analysis revealed that the combination treatment affected pathways associated with “Cell replication”, “DNA repair,” and “negative regulation of kinase phosphorylation,” all established hallmarks of the anti‐tumor effects of MAPK pathway inhibition (Bahar et al. 2023). To examine the effects of monotherapy treatment in the individual cell lines as compared to combination treatment, we visualized GO_BP for the individual treatments in a Venn diagram including comparison of combined treatment to vehicle (ctr) or single treatments (ST) (Figures S3A and S4A). We found that cell line M161227, which is sensitive to MAPKi, accumulated GO terms related to RNA processing and splicing, as well as DNA repair and cell division (mitosis), when treated with brimarafenib alone, while mirdametinib specifically induced DNA replication (Figure S3B,C). In combination, the dual inhibition of MAPK signaling—compared to the individual treatments—led to the downregulation of metabolic pathways. This is consistent with previous findings for BRAFi in BRAF‐mutated melanoma, where MAPK signaling can directly activate glycolysis (Figure S3D) (Shen et al. 2025). In parallel, we observed additional inhibition of cholesterol and steroid metabolism, which depends on glycolysis‐derived Acetyl‐CoA. Compared to the vehicle (ctrl), the combination treatment caused strong suppression of the cell cycle, DNA replication, and telomere stabilization. (Figure S3E). In contrast, the MEKi‐resistant melanoma cell line M130227 upregulated pathways related to “lipoprotein metabolism,” “long‐chain fatty acid metabolism,” and “sterol and steroid biosynthesis” when treated with either brimarafenib or mirdametinib alone (Figure S4B,C). This metabolic activation of fatty acid pathways in resistant cells in response to MAPKi has been reported previously by our group and others. (Aloia et al. 2019; Shen et al. 2020). Additionally, single‐drug treatments downregulate processes such as “double‐strand break repair via non‐homologous end joining” and “telomere maintenance via recombination” in response to brimarafenib. Mirdametinib, in turn, downregulates processes including “DNA‐templated transcription,” “regulation of translation,” and “ribosomal large subunit assembly,” which are important but not essential for cell proliferation. In contrast, the combination treatment induces a pronounced shutdown of critical cell division processes, including “chromosome condensation” and “establishment of mitotic spindle orientation,” highlighting its stronger impact on mitotic progression. (Figure 4D). Compared to the vehicle treatment (ctr) we observe an upregulation of “apoptotic signaling pathways” and “autophagy assembly” pointing towards an induction of apoptotic pathways. Significantly downregulated pathways by the combination treatments involve metabolic processes like “mitochondrial respiratory chain complex I assembly”, “aerobic respiration” and “nucleobase‐containing compound metabolic processes” as well as DNA damage regulation like “regulation of DNA damage checkpoint” and “DNA damage checkpoint signaling.” Overall, these data show that the MAPK pathway regulates different cellular processes depending on the context, and that dual inhibition is required to fully trigger the diverse effects of MAPK signaling.

### 3.4 Paradoxical Activation of MEK and ERK Are Antagonized by Brimarafenib and Mirdametinib Combination Treatment and Result in the Induction of Apoptosis

Monotherapies targeting MAPK pathway by MEK inhibitors can result in pathway activation. Previous reports show that inhibition of MEK kinase by MEK inhibitors like selumetinib in RAS mutated cancer cell lines triggers reactivation of CRAF leading to MEK binding, phosphorylation, and a decrease in sensitivity to MEK inhibitors (Lito et al. 2014). Conversely, RAF dimer inhibitor monotherapy (e.g., LY3009120) results in paradoxical ERK activation in BRAF WT tumor cells due to the formation of RAF heterodimers (Lito et al. 2014; Peng et al. 2015; Lai et al. 2022). Of note, a different RAF inhibitor, lifirafenib, was reported to induce MEK inhibition through another mechanism. Binding of RAF molecules (e.g., CRAF) to MEK kinase is induced by lifirafenib, resulting in inhibition of MEK but the induction of ERK phosphorylation (Yuan et al. 2020). Collectively, these paradoxical pathway effects mitigate the potential benefits of monotherapy treatment and, therefore, imply that the combination of a MEKi and RAF dimer inhibitor can compensate for the shortcomings of each monotherapy.

**FIGURE 5:**
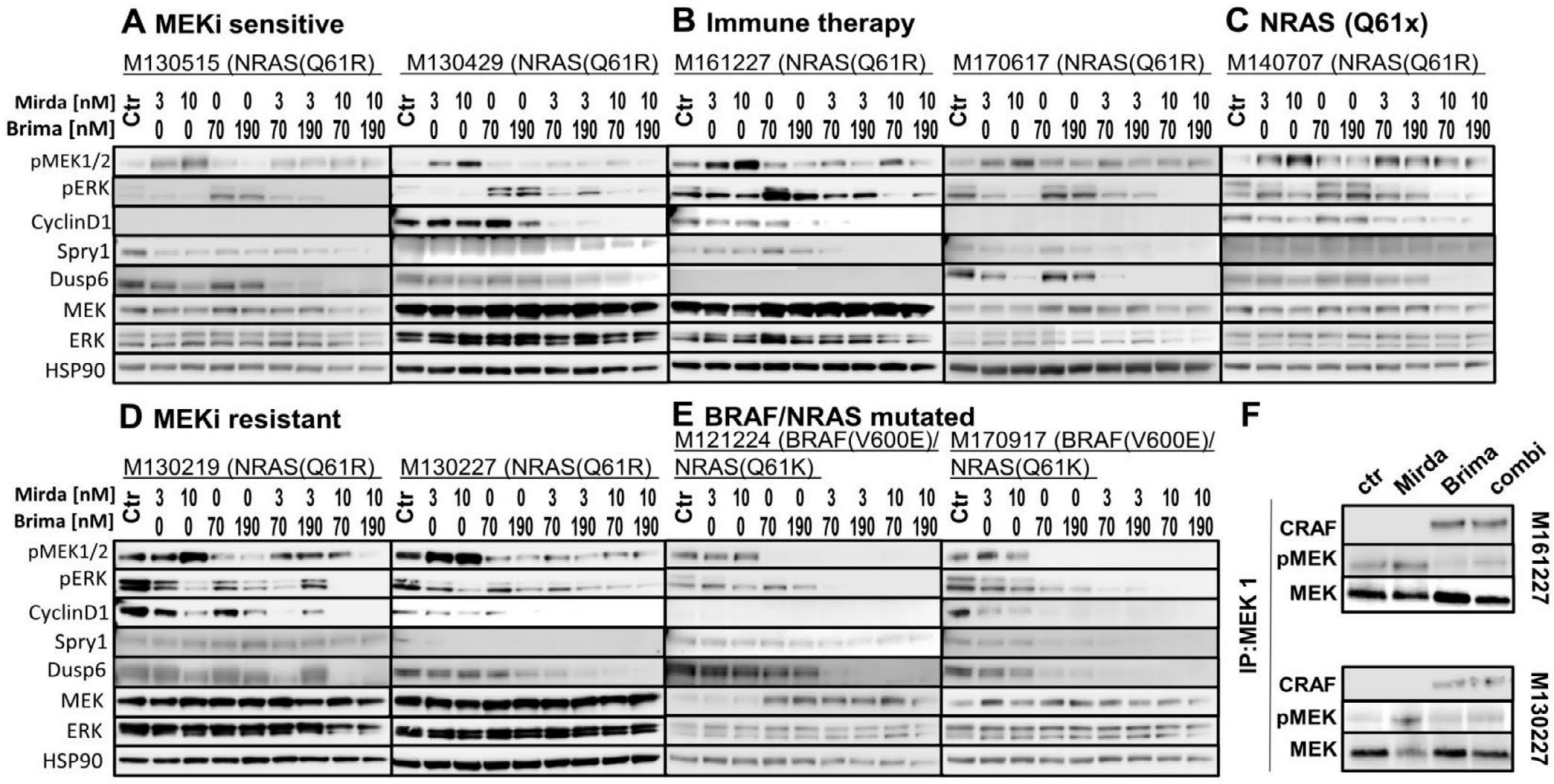
Dose‐dependent inhibition of the MAPK pathway with brimarafenib and mirdametinib treatment. (A–E) Patient‐derived melanoma cell lines were treated with 70 or 190 nM brimarafenib, 3 or 10 nM mirdametinib, or the combination for 24 h before cells were lysed and analyzed by Western blot for pMEK1/2, pERK, CCND1, SPRY1, and DUSP6. (F) Pull‐down experiments with antibodies binding to MEK1 kinase. Pull‐down eluates were probed with antibodies for CRAF, pMEK, and total MEK.

To interrogate the effects of brimarafenib and mirdametinib on MAPK protein expression, analysis of phosphorylated MEK and ERK, and the MAPK target genes CCND1, SPRY1 and DUSP6 were conducted by Western blot after treatment for 24 h (Figure 5A–E). In NRAS‐mutated melanoma cell lines, mirdametinib treatment led to increased MEK phosphorylation, though ERK phosphorylation was reduced in a dose‐dependent manner.

Treatment with brimarafenib alone downregulated pMEK protein levels, but increased pERK, and therefore had little to no impact on the expression of downstream MAPK target genes (Figure 5A–D). Only the combination of brimarafenib and mirdametinib effectively inhibited the upregulation of phosphorylated MEK, decreased phosphorylation of ERK and decreased expression of the MAPK target genes, independent of basal expression levels. Importantly, MAPK target genes were heterogeneously expressed between cell lines, and targets genes were not always expressed simultaneously, suggesting that other mechanisms may regulate protein expression and stability. The only exception was seen in cell lines derived from resistant patients, whose tumors harbored an initial BRAF V600E mutation and subsequently gained an NRAS mutation after developing resistance to BRAFi (Figure 5E). Treatment with brimarafenib resulted in reduced ERK protein phosphorylation. In BRAF V600E mutated melanoma, where activation of the MAPK pathway is due to the mutated BRAF protein only, treatment with a MEKi does not lead to reactivation of MEK through CRAF (Lito et al. 2014). Similar to observations in BRAF V600E mutated melanoma, no increase in pMEK was observed in M121224 and M170917 after mirdametinib treatment alone. This suggests that the BRAF V600E mutation is preventing the binding of MEK and CRAF. Furthermore, only brimarafenib and mirdametinib combination treatment prevented ERK phosphorylation and inhibited target gene expression. (Figure 5E).

In addition, treatment with brimarafenib triggered the binding of MEK to CRAF in the absence of MEK phosphorylation (Figure 5F). Mirdametinib treatment, however, increased the accumulation of phosphorylated MEK kinase (pMEK) but did not result in binding of MEK to CRAF (Figure 5F).

**FIGURE 6:**
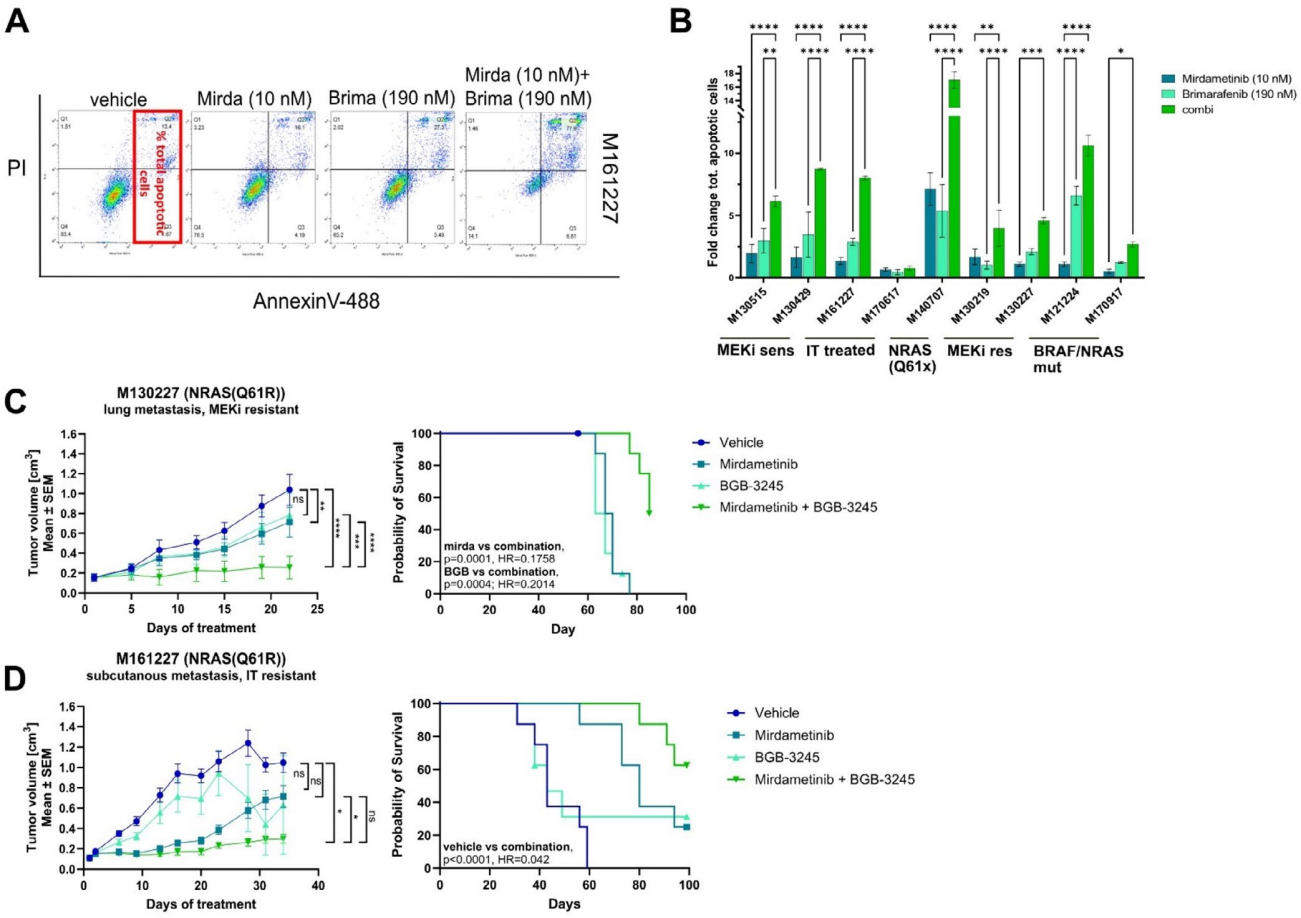
Induction of apoptosis in vitro and inhibition of tumor growth in vivo with the combination of brimarafenib and mirdametinib. (A) Representative example from a melanoma cell line (M161227), illustrating the gating strategy and results of flow cytometry analysis using Annexin V/PI staining. (B) All nine cell lines were treated for 72 h and harvested for flow cytometry analysis after staining with Annexin V‐488 and PI. Fold change is depicted in relation to vehicle (0.1% DMSO) treated control wells. Experiments were performed in duplicates and data were plotted using GraphPad Prism software (p* < 0.05, p** < 0.01, p*** < 0.001, p**** < 0.0001, two‐way ANOVA). (C, D) Left panel: Balb/c nude mice were subcutaneously implanted with 5 × 10^5^ cells derived from patients M130227 and M161227. Once tumors reached 0.1 cm^3^, mice were randomized and treated with either mirdametinib (0.5 mg/kg for M130227 or 0.3 mg/kg for M161227, BID), brimarafenib (2 mg/kg for M130227 or 1.5 mg/kg for M161227), or the combination. Data was analyzed using a Turkey's multiple comparisons test (repeated measures two‐way ANOVA). Right panel: Kaplan–Meier survival curves of mice implanted with M130227 and M161227 tumors. Statistics are shown only for the significant groups.

Activation of CRAF via phosphorylation was also observed in A375 resistant melanoma cells compared to the sensitive parental cell lines (Whittaker et al. 2015). Here, treatment of a combination of RAF and MEK inhibitors did induce greater suppression of MAPK pathway activity and increased apoptosis. PanRAF/MEKi combinations have generally been shown to potentiate apoptotic effects (Atefi et al. 2015). Consistently, we observed that combination therapy significantly altered the regulation of pro‐ and anti‐apoptotic factors (Figure 4C). To further investigate this, we assessed the effects of single‐agent and combination treatments on apoptosis using Annexin V detection on the cell surface of melanoma cells by flow cytometry (Figure 6A,B) and PARP cleavage by Western blotting (Figure S5C). We employed Annexin V and propidium iodide (PI) double staining to measure the total apoptotic fraction in each cell line (gating strategy shown in Figure 6A). In all cell lines, combination treatment significantly increased the proportion of apoptotic cells (Figure 6B). In agreement with the apoptotic assessments, significant correlative decreases in viable cell frequencies were seen across tested melanoma lines after combination treatment (Figure S5B). Moreover, the combination therapy induced PARP cleavage, particularly in MEKi‐sensitive cell lines, with the cleaved protein band detectable in all cell lines. (Figure S5C). To assess the potential mechanistic differences between monotherapy and combination treatment, cell cycle analysis was performed on the representative model M161227 (Figure S5A). This cell line is sensitive to mirdametinib (IC50 = 13 nM) and was arrested in G0‐G1 cell cycle phase when treated with brimarafenib or mirdametinib alone. Furthermore, in this cell line, low detectable PARP cleavage and minor fold‐changes in apoptosis were detected after monotherapy treatment, suggesting either monotherapy induces a primarily cytostatic effect. Vertical inhibition of the MAPK pathway by combination treatment induced robust cytotoxic effects and significantly increased induction of subG1 (Figure S5A,B).

Taken together, dual inhibition of the MAPK pathway with the panRAF inhibitor brimarafenib and the MEK inhibitor mirdametinib reduces phosphorylation of MEK and ERK and effectively induces apoptosis in melanoma cells.

### 3.5 Brimarafenib and Mirdametinib Combination Therapy Inhibit Tumor Growth and Prolong the Survival of Melanoma‐Bearing Mice

The combination therapy's efficacy was further evaluated in xenograft models using two patient‐derived melanoma cell lines, M130227 and M161227. Dosing of brimarafenib and mirdametinib was extrapolated from the clinical studies in humans and tumor growth was tracked until no mice were left in the vehicle treated group (Figure 6C,D, Tables S4 and S5). The monotherapy as well as the combination treatment was well tolerated by the animals (Figure S6).

Starting at Day 8 of dosing a significant treatment response relationship to combination therapy was seen in the M130227 model (*p* = 0.002) and the monotherapy treatment groups (mirdametinib, *p* = 0.0013; brimarafenib, *p* = 0.0007) (Figure 6C, Table S1). The magnitude of this effect increased until treatment Day 22with inhibition of tumor growth by the combination therapy compared to vehicle (*p* < 0.0001) or to the individual monotherapies (mirdametinib, *p* < 0.0001; brimarafenib, *p* = 0.0005) showing significant differences.

In correlation, average tumor volumes were compared between mirdametinib monotherapy and combination therapy recipients on study Day 63, the day vehicle treated control animals were terminated. Significantly smaller tumor volumes were seen in combination therapy recipients, highlighting the superior effect of combination therapy over monotherapy after long‐term treatment. (Figure S6A, Table S4) (*p* < 0.0001).

Additionally, analysis of individual mouse survival showed significant differences between treatment groups using log‐rank Matel‐Cox analysis. Mice treated with combination therapy lived significantly longer than mice treated with the individual monotherapy (mirdametinib vs. combination, *p* = 0.0001, Hazard ratio (HR) = 0.1758; brimarafenib vs. combination, *p* = 0.0004; HR = 0.2014). No significant difference in survival was seen after comparing vehicle treated animals with those receiving either monotherapy (Table S4). Specifically, mice treated with mirdametinib had a median survival of 68.5 days, whereas mice treated with brimarafenib had a median survival of 65 days. Mice treated with the combination treatment had a median survival of > 85 days, with 7/8 mice still alive at study termination, highlighting a clear survival benefit in mice receiving combination therapy.

For the M161227 tumor model, treatment with mirdametinib showed significant anti‐tumor effects compared to vehicle control recipients until treatment Day 28 (*p* = 0.009), after which tumor re‐growth was observed. In conjunction, significant differences in tumor inhibition were noted in combination therapy recipients starting on treatment Day 28 compared to mirdametinib monotherapy recipients (mirdametinib vs. combination *p* = 0.0213, Day 28). (Figure 6H, Table S5). Of note, significant differences in tumor inhibition between mirdametinib monotherapy and combination therapy recipients continued until Day 56 (Figure S5D). In the survival analyses with the Log‐rank Mantel‐Cox tests, significant differences were seen. Specifically, treatment with mirdametinib alone or combination treatment showed significantly longer survival when compared to the vehicle control group (mirdametinib [*p* < 0.0004, HR = 0.069] and combination [*p* < 0.0001, HR = 0.042]). Animals receiving brimarafenib monotherapy did not significantly survived longer compared to the vehicle treated group (*p* = 0.53, HR = 0.23). The median survival of both the vehicle‐treated and the brimarafenib monotherapy group was 43 days. By contrast, mice treated with mirdametinib had a median survival of 80 days, while combination treatment recipients had a median survival > 98 days, with five mice reaching study end while harboring smaller tumors. All these factors correlated with a clear benefit in survival after combination treatment.

Additionally, analysis of phosphorylated ERK protein was assessed after treatment in both xenograft studies. For these pharmacodynamic assessments, mice were treated three times within 24 h after tumors reached approximately 0.5 cm^3^. The tumors were excised 1 h after the last treatment and analyzed by immunohistochemistry for pERK (Figure S6B,C,E,F). The staining intensity was determined by the QuPath software using a semi‐quantitative score (weak = 1+, moderate = 2+, strong = 3+). In the slower growing M130227 tumors, 60% of all tumor cells stained positive for pERK (33% = 1+; 13% = 2+; 15% = 3+), whereas in the rapidly growing M161227 tumors, 90% of all tumor cells stained positive for pERK (30% = 1+; 28% = 2+; 32% = 3+), in vehicle treated tumors. After treatment, the overall frequency of pERK positive cells in M130227 tumors were not significantly reduced compared to vehicle control but the frequency of the highest intensity pERK stained cells (cells scored as 3+) were significantly reduced after combination treatment (*p* = 0.005). In M161227 tumors, a significant reduction in the frequency of pERK positive cells was seen after treatment with mirdametinib or combination therapy (*p* < 0.0001). Overall, the combination of brimarafenib and mirdametinib significantly reduced tumor growth, prolonged the survival of mice, and decreased activated MAPK signaling in the tumor, as measured by pERK.

## 4 Discussion

Melanomas are notoriously dependent on oncogenic mutations in the MAPK signaling pathway, making that pathway a favorable target in the treatment of metastatic disease. For example, BRAF inhibitors have been commonly used to treat melanomas, showing selective effects in tumors with BRAF V600 mutations. Moreover, combination therapy using BRAF inhibitors in conjunction with inhibitors targeting the down‐stream protein MEK has been proven to significantly prolong the overall survival of melanoma patients with BRAF V600 mutations, which represents about 50% of all melanoma cases (Ascierto et al. 2020). In contrast, in patients whose tumors harbor the expression of wild‐type BRAF or amplified MAPK pathway expression through RAS mutations, treatment with BRAF V600 inhibitors are contraindicated due to lack of efficacy. FDA‐approved BRAF V600 inhibitors promote RAS‐GTP driven Erk activation mainly attributed due to the selectivity of the first generation BRAF inhibitors for monomeric BRAF and the inability to alter formation of wild‐type RAF homo‐ and heterodimers leading to Erk phosphorylation (Halaban et al. 2010). Drug‐induced transactivation of RAF dimers underlies the paradoxical activation of the kinase by RAF inhibitors. Activation of ERK signaling occurs when the inhibitor binds to the ATP‐binding site of one kinase within the dimer, a process that depends on RAS activity. In RAF homodimers (CRAF–CRAF) or heterodimers (CRAF–BRAF), drug binding suppresses the activity of the targeted protomer but simultaneously triggers activation of the unbound partner protomer (Poulikakos et al. 2010; Kaplan et al. 2011). This mechanism is promoted not only by classical oncogenic RAS activity but also effected by unconventional MRAS activity in complex with SHOC2, a positive regulator of the Erk pathway, which leads to CRAF activation (Lai et al. 2022; Vasta et al. 2023). Therefore, the introduction of a novel class of RAF dimer inhibitors that also inhibit RAF dimerization was developed. Type II RAF inhibitors, like naporafenib and belvarafenib, have been tested in the clinic, but have only shown limited anti‐tumor activities as monotherapy. While these RAF dimer inhibitors do prevent the dimerization of BRAF and CRAF effectively, the inhibition towards ARAF is limited and alterations in ARAF protein can lead to resistance (Janku et al. 2024; Yen et al. 2021). Additionally, targeting NRAS‐mutant melanoma, strategies have centered on downstream MAPK blockade because direct pharmacologic inhibition of mutant NRAS has remained intractable. Single‐agent MEK inhibitors such as binimetinib, pimasertib, and others have demonstrated objective responses but generally modest durability: in the phase III NEMO trial, binimetinib improved PFS over dacarbazine yet failed to extend overall survival and incurred meaningful toxicity, while earlier‐phase data showed ~20% response rates and short PFS intervals. (Dummer et al. 2017; Salzmann et al. 2022). More recently, the MEK inhibitor tunlametinib achieved a 35.8% ORR with median PFS 4.2 months in a phase II study, suggesting incremental gains but persistent limitations (Wei et al. 2024). MEK inhibitors can drive the association of MEK kinase with RAF isoforms, which in turn leads to low binding affinity of MEK inhibitors. This phenomenon has been shown to be prevented by silencing RAF signaling (Zaremba et al. 2023). Of note, activation of CRAF has been demonstrated when RAS mutated tumor cells are treated with MEK or Erk inhibitors alone, resulting in the loss of a feedback control mechanism. Preclinical work in large NRAS‐mutant melanoma panels demonstrated that concurrent panRAF and MEK inhibition (e.g., Amgen compound A + trametinib) yields broad synergy, deeper pathway suppression, and apoptosis in MAPK‐dependent models; complementary functional screens implicate CRAF as a central bypass driver and show dual panRAF/MEK blockade can overcome diverse resistance mechanisms (Whittaker et al. 2015; Atefi et al. 2015). Mechanistic studies reveal that relief of ERK‐mediated negative feedback under MEK blockade drives CRAF‐dependent MAPK reactivation and recruitment of parallel survival pathways, fostering rapid resistance. Knock‐down of CRAF via siRNA, however, did improve suppression of downstream signaling when co‐administered with a MEK inhibitor (Lito et al. 2014). Translationally, the panRAF inhibitor naporafenib combined with trametinib produced preliminary antitumor activity in a phase Ib expansion cohort of previously treated NRAS‐mutant melanoma, catalyzing the ongoing SEACRAFT‐2 pivotal phase III trial (NCT06346067) (de Braud et al. 2023; Erasca 2025). Collectively, these data provide a strong biologic and clinical rationale for developing panRAF + MEK combinations to mitigate the limitations of monotherapy and result in more complete MAPK pathway inhibition in NRAS mutated melanoma tumors.

In this study we demonstrate that the combination of the investigational selective small molecule inhibitor of monomer and dimer forms of RAF, brimarafenib, in combination with the investigational selective and non‐ATP comparative MEK inhibitor mirdametinib, shows additive anti‐tumor efficacy in vitro and in vivo. Correlation analysis of killing curves after single drug or combination treatment revealed a significant additive effect for the combination therapy in all tested patient derived NRAS mutated melanoma cell lines in vitro. In general, additive effects of combination treatment are classified as effective and is supported by real world data. Specifically, data analysis using clinical trial information from the US FDA across the last 25 years on cancer drugs showed that 95% of clinical combination therapies are additive and that synergistic drug effects, in the sense of mathematical modelling, do not correlate with more efficacy in clinical trials (Hwangbo et al. 2023). Therefore, our observation of significant additive effects for the combination of brimarafenib and mirdametinib in in vitro assays prompted further investigation for the combination in a clinical trial (NCT05580770) (SpringWorks Therapeutics 2025). To evaluate the ability of combination therapy to prevent rebound to MEK or RAF dimer inhibitor monotherapy treatment, this clinical combination was assessed in long‐term colony formation assays. Colony formation assays enable the investigation of minimal residual disease, which clinically leads to relapse and resistance, and is crucial for RAS mutated melanoma based on publications citing rapid resistance to MAPK pathway inhibition. Vertical targeting of MEK and Erk kinases have already been shown to enhance therapeutic effects in in vitro *colony* assays (Rebecca et al. 2014). In line, significant inhibition of colony formation was seen with the combination of brimarafenib and mirdametinib in all patient‐derived melanoma cell lines. Furthermore, removal of drug treatment, which allows for colony regrowth, showed marked differences supportive of a higher degree of tumor cell suppression after combination therapy. In addition, in two different xenograft mouse models harboring tumors from NRAS mutated patient‐derived melanoma cells combination therapy showed superior anti‐tumor effects compared to animals administered either monotherapy, resulting in significant survival advantages. Along with this observation in vivo, RNAseq experiments showed that MAPK target genes are significant downregulated with the combination treatment. In contrast, gene expression differences after monotherapy treatment were variable in nature, suggesting that compensatory mechanisms are still active. Given the complex regulation of the MAPK pathway this is not surprising. Collectively, although melanoma cell lines may vary in response to MAPK inhibitors and the magnitude of changes in cytotoxic markers across assays, the combination of brimarafenib and mirdametinib induced significant cytotoxicity and correlative loss in viable cells compared to monotherapy, supporting the additive value of combination therapy.

Interestingly, the results of this study suggest a different mechanism by which these inhibitors function compared to other molecules. Treatment with mirdametinib did not provoke the binding of MEK kinase to CRAF, but rather induced MEK kinase phosphorylation and subsequent downregulation of ERK phosphorylation. It is hypothesized that this is due to loss of Erk‐driven negative feedback mechanisms and the inhibitory effects on the translocation of phosphorylation from MEK kinase to downstream targets by mirdametinib, therefore accumulating phosphorylation of MEK kinase. Moreover, the effects of brimarafenib were similar to published data for lifirafenib, which showed induced binding of MEK kinase to CRAF, thereby inhibiting MEK kinase phosphorylation while allowing ERK kinase phosphorylation (Yuan et al. 2020). Therefore, only the addition of both inhibitors induced effective inhibition of MAPK pathway signaling. Although the specific mechanism of brimarafenib and mirdametinib therapy may be unique, the combination provided significant benefit in representative in vivo models, prolonging overall survival and showing superior inhibition of tumor growth compared to monotherapy treatment. A clinical trial is currently recruiting patients with solid tumors to assess the combination of brimarafenib and mirdametinib (NCT05580770) (SpringWorks Therapeutics 2025). Overall, the results of this study highlight the benefit of brimarafenib and mirdametinib combination treatment in NRAS mutated melanoma models and provide supportive preclinical evidence for the investigation of this combination approach in melanoma patients harboring NRAS or other RAS‐mutations.

## Supporting information


**Table S1:** Differentially expression analysis (DEseq) between Mirda (mirdametinib)_BGB (brimarafenib) combination and vehicle (DMSO), Mirda (mirdametinib) versus DMSO, BGB (brimarafenib) versus DMSO, Mirda_BGB versus DMSO in melanoma cell line M130227.

**Table S2:** Differentially expression analysis (DEseq) between Mirda (mirdametinib)_BGB (brimarafenib) combination and vehicle (DMSO), Mirda (mirdametinib) versus DMSO, BGB (brimarafenib) versus DMSO, Mirda_BGB versus DMSO in melanoma cell line M161227.

**Table S3:** Combined differentially expression analysis (DEseq) between Mirda (mirdametinib)_BGB (Brimarafenib) combination and vehicle (DMSO) for both melanoma cell lines (M130227 and M161227).

**Table S4:** Gene set enrichment analysis for GO_Biological Processes using RNAseq data (DEseq) from cell line M130227.

**Table S5:** Gene set enrichment analysis for GO_Biological Processes using RNAseq data (DEseq) from cell line M161227.

**Table S6:** Statistical analysis of data from the in vivo Xenograft experiment performed with patient‐derived cell line M130227.

**Table S7:** Statistical analysis of data from the in vivo Xenograft experiment performed with patient‐derived cell line M161227.

**Figure S1:** Dose‐dependent growth inhibition and IC_50_ summary for mirdametinib and brimarafenib. (A) Individual growth inhibition curves for patient‐derived melanoma cell lines treated with increasing concentrations of mirdametinib or brimarafenib. Data represent the mean of technical triplicates from two independent experiments. Curves and IC_50_ values were generated using GraphPad Prism software. (B) Summary of IC_50_ values (nM) for binimetinib, mirdametinib, and brimarafenib, either alone or in combination. IC_50_ values were derived from the dose–response data shown in Figure 1A–E.
**Figure S2:** Quantification of colony formation following treatment with brimarafenib and mirdametinib. (A–E) Bar graphs showing the percentage intensity output from the colony formation assay (CFA) corresponding to the experiment presented in Figure 2. Each bar represents the mean of two independent experiments, each performed in technical triplicate.(*p* < 0.05, *p < 0.01, **p < 0.001, ***p < 0.0001; two‐way ANOVA). Vehicle = control wells treated with 0.2% DMSO, M3 = mirdametinib (3 nM), M10 = mirdametinib (10 nM), B70 = brimarafenib (70 nM), B190 = brimarafenib (190 nM), M3_B70 = mirdametinib (3 nM) + binimetinib (70 nM), M10_B70 = mirdametinib (10 nM) + binimetinib (70 nM), M3_B190 = mirdametinib (3 nM) + binimetinib (190 nM), M10_B190 = mirdametinib (10 nM) + binimetinib (190 nM).
**Figure S3:** Gene Set Enrichment Analysis of M161227 following treatment with brimarafenib and mirdametinib. (A) Venn diagram comparing enriched GO Biological Processes (see Table S5) in cell line M161227 for the following comparisons: brimarafenib versus control (BGB vs. ctr), mirdametinib versus control (Mirda vs. ctr), combination versus vehicle control (0.2% DMSO) (BGB_Mirda vs. ctr), and combination versus single treatments (BGB_Mirda vs. ST). (B–E) GO Biological Processes exclusive to each comparison, as identified in the Venn diagram, are shown as bar graphs. Bar height represents the normalized enrichment score (NES), and bars are color‐coded according to adjusted *p*‐value. Plots were generated using the R package ggplot2 (Wickham 2016).
**Figure S4:** Gene Set Enrichment Analysis of M130227 following treatment with brimarafenib and mirdametinib. (A) Venn diagram comparing enriched GO Biological Processes (see Table S4) in cell line M130227 for the following comparisons: brimarafenib versus control (BGB vs. ctr), mirdametinib versus control (Mirda vs. ctr), combination versus vehicle control (0.2% DMSO) (BGB_Mirda vs. ctr), and combination versus single treatments (BGB_Mirda vs. ST). (B–E) GO Biological Processes exclusive to each comparison, as identified in the Venn diagram, are shown as bar graphs. Bar height represents the normalized enrichment score (NES), and bars are color‐coded according to adjusted *p*‐value. Plots were generated using the R package ggplot2 (Wickham 2016).
**Figure S5:** Cell cycle analysis and apoptosis induction following treatment with mirdametinib and brimarafenib. (A) Cell cycle distribution analysis of M161227 assessed by flow cytometry using propidium iodide (PI) staining. Cells were treated for 72 h with indicated compounds (M (10 nM) = mirdametinib 10 nM; B (90 nM) = brimarafenib 90 nM) or vehicle (0.2% DMSO). Data represent the summary of three independent experiments. Statistics was performed on cell cycle phases indicated (p* < 0.05, p*** < 0.001, p**** < 0.0001, two‐way ANOVA). (B) Frequency of viable melanoma cells after treatment with mirdametinib (30 nM), brimarafenib (100 nM) or the combination normalized to vehicle control (0.2% DMSO). (p* < 0.05, p** < 0.01, p*** < 0.001, p**** < 0.0001, two‐way ANOVA). (C) Western blot analysis of PARP cleavage following treatment with various concentrations of mirdametinib, brimarafenib, and their combination.
**Figure S6:** Tumor volume and immunohistochemical analysis of xenograft tumors. (A, D) Tumor volumes for individual mice on the final day of treatment (Day 63 for M130227 and Day 56 for M161227) following mirdametinib or combination therapy. (B,E) Representative immunohistochemistry images of pERK and S100 staining in xenograft tumor tissues (B: M130227, E: M161227). (C,F) Quantification of immunohistochemistry staining intensities using QuPath software for M130227 (C) and M161227 (F). Staining intensities were automatically classified using threshold levels of 0.1 = 1+ (weak), 0.2 = 2+ (moderate), and 0.4 = 3+ (strong). Statistics performed on 3+ scoring (p*** < 0.001, p**** < 0.0001, two‐way ANOVA).
**Figure S7:** Body weight of animals measured during the in vivo experiments.

## Data Availability

The data that supports the findings of this study are available in the Supporting Information of this article.

## References

[pcmr70062-bib-0001] Aloia, A. , D. Müllhaupt , C. D. Chabbert , et al. 2019. “A Fatty Acid Oxidation‐Dependent Metabolic Shift Regulates the Adaptation of BRAF‐Mutated Melanoma to MAPK Inhibitors.” Clinical Cancer Research 25, no. 22: 6852–6867. 31375515 10.1158/1078-0432.CCR-19-0253 PMC6906212

[pcmr70062-bib-0002] Ascierto, P. A. , R. Dummer , H. J. Gogas , et al. 2020. “Update on Tolerability and Overall Survival in COLUMBUS: Landmark Analysis of a Randomised Phase 3 Trial of Encorafenib Plus Binimetinib vs Vemurafenib or Encorafenib in Patients With BRAF V600‐Mutant Melanoma.” European Journal of Cancer 126: 33–44. 31901705 10.1016/j.ejca.2019.11.016

[pcmr70062-bib-0003] Atefi, M. , B. Titz , E. Avramis , et al. 2015. “Combination of Pan‐RAF and MEK Inhibitors in NRAS Mutant Melanoma.” Molecular Cancer 14, no. 1: 27. 25645078 10.1186/s12943-015-0293-5 PMC4320814

[pcmr70062-bib-0004] Bahar, M. E. , H. J. Kim , and D. R. Kim . 2023. “Targeting the RAS/RAF/MAPK Pathway for Cancer Therapy: From Mechanism to Clinical Studies.” Signal Transduction and Targeted Therapy 8, no. 1: 455. 38105263 10.1038/s41392-023-01705-z PMC10725898

[pcmr70062-bib-0005] Bankhead, P. , M. B. Loughrey , J. A. Fernández , et al. 2017. “QuPath: Open Source Software for Digital Pathology Image Analysis.” Scientific Reports 7, no. 1: 16878. 29203879 10.1038/s41598-017-17204-5 PMC5715110

[pcmr70062-bib-0006] Brix, N. , D. Samaga , C. Belka , H. Zitzelsberger , and K. Lauber . 2021. “Analysis of Clonogenic Growth In Vitro.” Nature Protocols 16, no. 11: 4963–4991. 34697469 10.1038/s41596-021-00615-0

[pcmr70062-bib-0007] Ci, C. , B. Tang , D. Lyu , et al. 2019. “Overexpression of CDCA8 Promotes the Malignant Progression of Cutaneous Melanoma and Leads to Poor Prognosis.” International Journal of Molecular Medicine 43, no. 1: 404–412. 30431060 10.3892/ijmm.2018.3985 PMC6257860

[pcmr70062-bib-0008] de Braud, F. , C. Dooms , R. S. Heist , et al. 2023. “Initial Evidence for the Efficacy of Naporafenib in Combination With Trametinib in NRAS‐Mutant Melanoma: Results From the Expansion Arm of a Phase Ib, Open‐Label Study.” Journal of Clinical Oncology 41, no. 14: 2651–2660. 36947734 10.1200/JCO.22.02018

[pcmr70062-bib-0009] Drosten, M. , and M. Barbacid . 2020. “Targeting the MAPK Pathway in KRAS‐Driven Tumors.” Cancer Cell 37, no. 4: 543–550. 32289276 10.1016/j.ccell.2020.03.013

[pcmr70062-bib-0010] Dry, J. R. , S. Pavey , C. A. Pratilas , et al. 2010. “Transcriptional Pathway Signatures Predict MEK Addiction and Response to Selumetinib (AZD6244).” Cancer Research 70, no. 6: 2264–2273. 20215513 10.1158/0008-5472.CAN-09-1577 PMC3166660

[pcmr70062-bib-0011] Dummer, R. , D. Schadendorf , P. A. Ascierto , et al. 2017. “Binimetinib Versus Dacarbazine in Patients With Advanced NRAS‐Mutant Melanoma (NEMO): A Multicentre, Open‐Label, Randomised, Phase 3 Trial.” Lancet Oncology 18, no. 4: 435–445. 28284557 10.1016/S1470-2045(17)30180-8

[pcmr70062-bib-0012] Eichhoff, O. M. , C. I. Stoffel , J. Käsler , et al. 2023. “ROS Induction Targets Persister Cancer Cells With Low Metabolic Activity in NRAS‐Mutated Melanoma.” Cancer Research 83, no. 7: 1128–1146. 36946761 10.1158/0008-5472.CAN-22-1826

[pcmr70062-bib-0013] Erasca, I. 2025. “A Study to Assess Naporafenib (ERAS‐254) Administered With Trametinib in Patients With NRAS‐Mutant Melanoma (SEACRAFT‐2).” ClinicalTrials.gov.

[pcmr70062-bib-0014] Franken, N. A. , H. M. Rodermond , J. Stap , J. Haveman , and C. van Bree . 2006. “Clonogenic Assay of Cells In Vitro.” Nature Protocols 1, no. 5: 2315–2319. 17406473 10.1038/nprot.2006.339

[pcmr70062-bib-0066] Freiberger, S. N. , G. B. Morand , P. Turko , et al. 2019. “Morpho‐Molecular Assessment Indicates New Prognostic Aspects and Personalized Therapeutic Options in Sinonasal Melanoma.” Cancers (Basel) 11: 1329. 31500314 10.3390/cancers11091329 PMC6770844

[pcmr70062-bib-0015] Garrido, G. , and I. Vernos . 2016. “Non‐Centrosomal TPX2‐Dependent Regulation of the Aurora A Kinase: Functional Implications for Healthy and Pathological Cell Division.” Frontiers in Oncology 6: 88. 27148480 10.3389/fonc.2016.00088 PMC4831974

[pcmr70062-bib-0016] Guzman, C. , M. Bagga , A. Kaur , et al. 2014. “ColonyArea: An ImageJ Plugin to Automatically Quantify Colony Formation in Clonogenic Assays.” PLoS One 9, no. 3: e92444. 24647355 10.1371/journal.pone.0092444 PMC3960247

[pcmr70062-bib-0017] Halaban, R. , W. Zhang , A. Bacchiocchi , et al. 2010. “PLX4032, a Selective BRAF(V600E) Kinase Inhibitor, Activates the ERK Pathway and Enhances Cell Migration and Proliferation of BRAF Melanoma Cells.” Pigment Cell & Melanoma Research 23, no. 2: 190–200. 20149136 10.1111/j.1755-148X.2010.00685.x PMC2848976

[pcmr70062-bib-0018] Hayward, N. K. , J. S. Wilmott , N. Waddell , et al. 2017. “Whole‐Genome Landscapes of Major Melanoma Subtypes.” Nature 545, no. 7653: 175–180. 28467829 10.1038/nature22071

[pcmr70062-bib-0019] Hilbers, M. L. , R. Brändli , B. Mühleisen , S. N. Freiberger , J. Mangana , and R. Dummer . 2021. “Standardized Diagnostic Algorithm for Spitzoid Lesions Aids Clinical Decision‐Making and Management: A Case Series From a Swiss Reference Center.” Oncotarget 12, no. 2: 125–130. 33520116 10.18632/oncotarget.27854 PMC7825637

[pcmr70062-bib-0020] Hussein, M. R. , A. K. Haemel , and G. S. Wood . 2003. “Apoptosis and Melanoma: Molecular Mechanisms.” Journal of Pathology 199, no. 3: 275–288. 12579529 10.1002/path.1300

[pcmr70062-bib-0021] Hwangbo, H. , S. C. Patterson , A. Dai , D. Plana , and A. C. Palmer . 2023. “Additivity Predicts the Efficacy of Most Approved Combination Therapies for Advanced Cancer.” Nature Cancer 4, no. 12: 1693–1704. 37974028 10.1038/s43018-023-00667-z

[pcmr70062-bib-0022] Ianevski, A. , A. K. Giri , and T. Aittokallio . 2020. “SynergyFinder 2.0: Visual Analytics of Multi‐Drug Combination Synergies.” Nucleic Acids Research 48, no. W1: W488–w493. 32246720 10.1093/nar/gkaa216 PMC7319457

[pcmr70062-bib-0023] Janku, F. , T. M. Kim , G. Iyer , et al. 2024. “First‐In‐Human Study of Naporafenib (LXH254) With or Without Spartalizumab in Adult Patients With Advanced Solid Tumors Harboring MAPK Signaling Pathway Alterations.” European Journal of Cancer 196: 113458. 38039779 10.1016/j.ejca.2023.113458 PMC11380116

[pcmr70062-bib-0024] Joseph, E. W. , C. A. Pratilas , P. I. Poulikakos , et al. 2010. “The RAF Inhibitor PLX4032 Inhibits ERK Signaling and Tumor Cell Proliferation in a V600E BRAF‐Selective Manner.” Proceedings of the National Academy of Sciences of the United States of America 107, no. 33: 14903–14908. 20668238 10.1073/pnas.1008990107 PMC2930420

[pcmr70062-bib-0025] Kaplan, F. M. , Y. Shao , M. M. Mayberry , and A. E. Aplin . 2011. “Hyperactivation of MEK‐ERK1/2 Signaling and Resistance to Apoptosis Induced by the Oncogenic B‐RAF Inhibitor, PLX4720, in Mutant N‐RAS Melanoma Cells.” Oncogene 30, no. 3: 366–371. 20818433 10.1038/onc.2010.408 PMC6591715

[pcmr70062-bib-0026] Karras, P. , I. Bordeu , J. Pozniak , et al. 2022. “A Cellular Hierarchy in Melanoma Uncouples Growth and Metastasis.” Nature 610, no. 7930: 190–198. 36131018 10.1038/s41586-022-05242-7 PMC10439739

[pcmr70062-bib-0027] Lai, L. P. , N. Fer , W. Burgan , et al. 2022. “Classical RAS Proteins Are Not Essential for Paradoxical ERK Activation Induced by RAF Inhibitors.” Proceedings of the National Academy of Sciences of the United States of America 119, no. 5: e2113491119. 35091470 10.1073/pnas.2113491119 PMC8812530

[pcmr70062-bib-0028] Larkin, J. , P. A. Ascierto , B. Dréno , et al. 2014. “Combined Vemurafenib and Cobimetinib in BRAF‐Mutated Melanoma.” New England Journal of Medicine 371, no. 20: 1867–1876. 25265494 10.1056/NEJMoa1408868

[pcmr70062-bib-0029] Leonardi, G. C. , L. Falzone , R. Salemi , et al. 2018. “Cutaneous Melanoma: From Pathogenesis to Therapy (Review).” International Journal of Oncology 52, no. 4: 1071–1080. 29532857 10.3892/ijo.2018.4287 PMC5843392

[pcmr70062-bib-0030] Li, Y. , J. Wei , Y. Sun , et al. 2023. “DLGAP5 Regulates the Proliferation, Migration, Invasion, and Cell Cycle of Breast Cancer Cells via the JAK2/STAT3 Signaling Axis.” International Journal of Molecular Sciences 24, no. 21: 15819. 37958803 10.3390/ijms242115819 PMC10647495

[pcmr70062-bib-0031] Lito, P. , A. Saborowski , J. Yue , et al. 2014. “Disruption of CRAF‐Mediated MEK Activation Is Required for Effective MEK Inhibition in KRAS Mutant Tumors.” Cancer Cell 25, no. 5: 697–710. 24746704 10.1016/j.ccr.2014.03.011 PMC4049532

[pcmr70062-bib-0032] Long, G. V. , A. M. Menzies , A. M. Nagrial , et al. 2011. “Prognostic and Clinicopathologic Associations of Oncogenic BRAF in Metastatic Melanoma.” Journal of Clinical Oncology 29, no. 10: 1239–1246. 21343559 10.1200/JCO.2010.32.4327

[pcmr70062-bib-0033] Long, G. V. , D. Stroyakovskiy , H. Gogas , et al. 2014. “Combined BRAF and MEK Inhibition Versus BRAF Inhibition Alone in Melanoma.” New England Journal of Medicine 371, no. 20: 1877–1888. 25265492 10.1056/NEJMoa1406037

[pcmr70062-bib-0034] LoRusso, P. M. , S. S. Krishnamurthi , J. J. Rinehart , et al. 2010. “Phase I Pharmacokinetic and Pharmacodynamic Study of the Oral MAPK/ERK Kinase Inhibitor PD‐0325901 in Patients With Advanced Cancers.” Clinical Cancer Research 16, no. 6: 1924–1937. 20215549 10.1158/1078-0432.CCR-09-1883

[pcmr70062-bib-0035] Ma, Y. , J. Gan , Y. Bai , D. Cao , and Y. Jiao . 2023. “Minimal Residual Disease in Solid Tumors: An Overview.” Frontiers in Medicine 17, no. 4: 649–674. 10.1007/s11684-023-1018-6 37707677

[pcmr70062-bib-0036] McArthur, G. A. , P. B. Chapman , C. Robert , et al. 2014. “Safety and Efficacy of Vemurafenib in BRAF(V600E) and BRAF(V600K) Mutation‐Positive Melanoma (BRIM‐3): Extended Follow‐Up of a Phase 3, Randomised, Open‐Label Study.” Lancet Oncology 15, no. 3: 323–332. 24508103 10.1016/S1470-2045(14)70012-9 PMC4382632

[pcmr70062-bib-0037] Oliveros, J. C. 2007–2015. “An Interactive Tool for Comparing Lists With Venn's Diagrams.” Venny.

[pcmr70062-bib-0038] Pagani, E. , F. Ruffini , G. C. A. Cappellini , et al. 2016. “Placenta Growth Factor and Neuropilin‐1 Collaborate in Promoting Melanoma Aggressiveness.” International Journal of Oncology 48, no. 4: 1581–1589. 26846845 10.3892/ijo.2016.3362

[pcmr70062-bib-0039] Peng, S. B. , J. R. Henry , M. D. Kaufman , et al. 2015. “Inhibition of RAF Isoforms and Active Dimers by LY3009120 Leads to Anti‐Tumor Activities in RAS or BRAF Mutant Cancers.” Cancer Cell 28, no. 3: 384–398. 26343583 10.1016/j.ccell.2015.08.002

[pcmr70062-bib-0040] Poulikakos, P. I. , C. Zhang , G. Bollag , K. M. Shokat , and N. Rosen . 2010. “RAF Inhibitors Transactivate RAF Dimers and ERK Signalling in Cells With Wild‐Type BRAF.” Nature 464, no. 7287: 427–430. 20179705 10.1038/nature08902 PMC3178447

[pcmr70062-bib-0041] Raaijmakers, M. I. , D. S. Widmer , M. Maudrich , et al. 2015. “A New Live‐Cell Biobank Workflow Efficiently Recovers Heterogeneous Melanoma Cells From Native Biopsies.” Experimental Dermatology 24, no. 5: 377–380. 25739758 10.1111/exd.12683

[pcmr70062-bib-0042] Raaijmakers, M. I. , D. S. Widmer , A. Narechania , et al. 2016. “Co‐Existence of BRAF and NRAS Driver Mutations in the Same Melanoma Cells Results in Heterogeneity of Targeted Therapy Resistance.” Oncotarget 7, no. 47: 77163–77174. 27791198 10.18632/oncotarget.12848 PMC5363577

[pcmr70062-bib-0043] Rambow, F. , A. Rogiers , O. Marin‐Bejar , et al. 2018. “Toward Minimal Residual Disease‐Directed Therapy in Melanoma.” Cell 174, no. 4: 843–855. 30017245 10.1016/j.cell.2018.06.025

[pcmr70062-bib-0044] Rebecca, V. W. , G. M. Alicea , K. H. Paraiso , H. Lawrence , G. T. Gibney , and K. S. Smalley . 2014. “Vertical Inhibition of the MAPK Pathway Enhances Therapeutic Responses in NRAS‐Mutant Melanoma.” Pigment Cell & Melanoma Research 27, no. 6: 1154–1158. 25130256 10.1111/pcmr.12303 PMC4211982

[pcmr70062-bib-0045] Rebocho, A. P. , and R. Marais . 2013. “ARAF Acts as a Scaffold to Stabilize BRAF:CRAF Heterodimers.” Oncogene 32, no. 26: 3207–3212. 22926515 10.1038/onc.2012.330

[pcmr70062-bib-0046] Salzmann, M. , J. Pawlowski , C. Loquai , et al. 2022. “MEK Inhibitors for Pre‐Treated, NRAS‐Mutated Metastatic Melanoma: A Multi‐Centre, Retrospective Study.” European Journal of Cancer 166: 24–32. 35272084 10.1016/j.ejca.2022.02.008

[pcmr70062-bib-0047] Samatar, A. A. , and P. I. Poulikakos . 2014. “Targeting RAS‐ERK Signalling in Cancer: Promises and Challenges.” Nature Reviews. Drug Discovery 13, no. 12: 928–942. 25435214 10.1038/nrd4281

[pcmr70062-bib-0048] Schram, A. M. , V. Subbiah , R. Sullivan , et al. 2023. “Abstract CT031: A First‐In‐Human, Phase 1a/1b, Open‐Label, Dose‐Escalation and Expansion Study to Investigate the Safety, Pharmacokinetics, and Antitumor Activity of the RAF Dimer Inhibitor BGB‐3245 in Patients With Advanced or Refractory Tumors.” Cancer Research 83, no. 8_Supplement: CT031‐CT031.

[pcmr70062-bib-0049] Shen, D. , L. Zhang , S. Li , and L. Tang . 2025. “Metabolic Reprogramming in Melanoma Therapy.” Cell Death Discovery 11, no. 1: 308. 40617808 10.1038/s41420-025-02617-3 PMC12228840

[pcmr70062-bib-0050] Shen, S. , S. Faouzi , S. Souquere , et al. 2020. “Melanoma Persister Cells Are Tolerant to BRAF/MEK Inhibitors via ACOX1‐Mediated Fatty Acid Oxidation.” Cell Reports 33, no. 8: 108421. 33238129 10.1016/j.celrep.2020.108421

[pcmr70062-bib-0051] Shi, H. , W. Hugo , X. Kong , et al. 2014. “Acquired Resistance and Clonal Evolution in Melanoma During BRAF Inhibitor Therapy.” Cancer Discovery 4, no. 1: 80–93. 24265155 10.1158/2159-8290.CD-13-0642 PMC3936420

[pcmr70062-bib-0052] Solomon, B. , B. Gao , V. Subbiah , et al. 2023. “Abstract CT033: Safety, Pharmacokinetics, and Antitumor Activity Findings From a Phase 1b, Open‐Label, Dose‐Escalation and Expansion Study Investigating RAF Dimer Inhibitor Lifirafenib in Combination With MEK Inhibitor Mirdametinib in Patients With Advanced or Refractory Solid Tumors.” Cancer Research 83, no. 8_Supplement: CT033‐CT033.

[pcmr70062-bib-0053] SpringWorks Therapeutics . 2025. “Mirdametinib + BGB‐3245 in Advanced Solid Tumors.” ClinicalTrials.gov.

[pcmr70062-bib-0054] Team, R.C . 2023. “R: A Language and Environment for Statistical Computing.” R Foundation for Statistical Computing, Vienna, Austria.

[pcmr70062-bib-0055] Thumar, J. , D. Shahbazian , S. A. Aziz , L. B. Jilaveanu , and H. M. Kluger . 2014. “MEK Targeting in N‐RAS Mutated Metastatic Melanoma.” Molecular Cancer 13: 45. 24588908 10.1186/1476-4598-13-45 PMC3945937

[pcmr70062-bib-0056] Vasta, J. D. , A. Michaud , C. A. Zimprich , et al. 2023. “Protomer Selectivity of Type II RAF Inhibitors Within the RAS/RAF Complex.” Cell Chemical Biology 30, no. 11: 1354–1365. 37643616 10.1016/j.chembiol.2023.07.019

[pcmr70062-bib-0057] Vu, H. L. , and A. E. Aplin . 2016. “Targeting Mutant NRAS Signaling Pathways in Melanoma.” Pharmacological Research 107: 111–116. 26987942 10.1016/j.phrs.2016.03.007 PMC4867277

[pcmr70062-bib-0058] Wei, X. , Z. Zou , W. Zhang , et al. 2024. “A Phase II Study of Efficacy and Safety of the MEK Inhibitor Tunlametinib in Patients With Advanced NRAS‐Mutant Melanoma.” European Journal of Cancer 202: 114008. 38479118 10.1016/j.ejca.2024.114008

[pcmr70062-bib-0059] Wellbrock, C. , M. Karasarides , and R. Marais . 2004. “The RAF Proteins Take Centre Stage.” Nature Reviews. Molecular Cell Biology 5, no. 11: 875–885. 15520807 10.1038/nrm1498

[pcmr70062-bib-0060] Whittaker, S. R. , G. S. Cowley , S. Wagner , F. Luo , D. E. Root , and L. A. Garraway . 2015. “Combined Pan‐RAF and MEK Inhibition Overcomes Multiple Resistance Mechanisms to Selective RAF Inhibitors.” Molecular Cancer Therapeutics 14, no. 12: 2700–2711. 26351322 10.1158/1535-7163.MCT-15-0136-T PMC4674359

[pcmr70062-bib-0061] Wickham, H. 2016. “ggplot2: Elegant Graphics for Data Analysis.”

[pcmr70062-bib-0062] Yen, I. , F. Shanahan , J. Lee , et al. 2021. “ARAF Mutations Confer Resistance to the RAF Inhibitor Belvarafenib in Melanoma.” Nature 594, no. 7863: 418–423. 33953400 10.1038/s41586-021-03515-1

[pcmr70062-bib-0063] Yu, G. , L. G. Wang , Y. Han , and Q. Y. He . 2012. “clusterProfiler: An R Package for Comparing Biological Themes Among Gene Clusters.” OMICS 16, no. 5: 284–287. 22455463 10.1089/omi.2011.0118 PMC3339379

[pcmr70062-bib-0064] Yuan, X. , Z. Tang , R. du , et al. 2020. “RAF Dimer Inhibition Enhances the Antitumor Activity of MEK Inhibitors in K‐RAS Mutant Tumors.” Molecular Oncology 14, no. 8: 1833–1849. 32336014 10.1002/1878-0261.12698 PMC7400788

[pcmr70062-bib-0065] Zaremba, A. , P. Mohr , R. Gutzmer , et al. 2023. “Immune Checkpoint Inhibition in Patients With NRAS Mutated and NRAS Wild Type Melanoma: A Multicenter Dermatologic Cooperative Oncology Group Study on 637 Patients From the Prospective Skin Cancer Registry ADOREG.” European Journal of Cancer 188: 140–151. 37245442 10.1016/j.ejca.2023.04.008

