# Supplementary material for: Combination of the Novel RAF Dimer Inhibitor Brimarafenib With the MEK Inhibitor Mirdametinib Is Effective Against NRAS Mutant Melanoma
Source: Pigment Cell Melanoma Res. 2025 Nov 6;38(6):e70062. doi: 10.1111/pcmr.70062 (PMC12592225; doi:10.1111/pcmr.70062)
Supplement: Supplementary file 8 — Figure S1: Dose‐dependent growth inhibition and IC50 summary for mirdametinib and brimarafenib. (A) Individual growth inhibition curves for patient‐derived melanoma cell lines treated with increasing concentrations of mirdametinib or brimarafenib. Data represent the mean of technical triplicates from two independent experiments. Curves and IC50 values were generated using GraphPad Prism software. (B) Summary of IC50 values (nM) for binimetinib, mirdametinib, and brimarafenib, either alone or in combination. IC50 values were derived from the dose–response data shown in Figure 1A–E. Figure S2: Quantification of colony formation following treatment with brimarafenib and mirdametinib. (A–E) Bar graphs showing the percentage intensity output from the colony formation assay (CFA) corresponding to the experiment presented in Figure 2. Each bar represents the mean of two independent experiments, each performed in technical triplicate.(p < 0.05, *p < 0.01, **p < 0.001, ***p < 0.0001; two‐way ANOVA). Vehicle = control wells treated with 0.2% DMSO, M3 = mirdametinib (3 nM), M10 = mirdametinib (10 nM), B70 = brimarafenib (70 nM), B190 = brimarafenib (190 nM), M3_B70 = mirdametinib (3 nM) + binimetinib (70 nM), M10_B70 = mirdametinib (10 nM) + binimetinib (70 nM), M3_B190 = mirdametinib (3 nM) + binimetinib (190 nM), M10_B190 = mirdametinib (10 nM) + binimetinib (190 nM). Figure S3: Gene Set Enrichment Analysis of M161227 following treatment with brimarafenib and mirdametinib. (A) Venn diagram comparing enriched GO Biological Processes (see Table S5) in cell line M161227 for the following comparisons: brimarafenib versus control (BGB vs. ctr), mirdametinib versus control (Mirda vs. ctr), combination versus vehicle control (0.2% DMSO) (BGB_Mirda vs. ctr), and combination versus single treatments (BGB_Mirda vs. ST). (B–E) GO Biological Processes exclusive to each comparison, as identified in the Venn diagram, are shown as bar graphs. Bar height represents the normalized enrichment [file PCMR-38-0-s005.docx]

**Supplementary Figures**


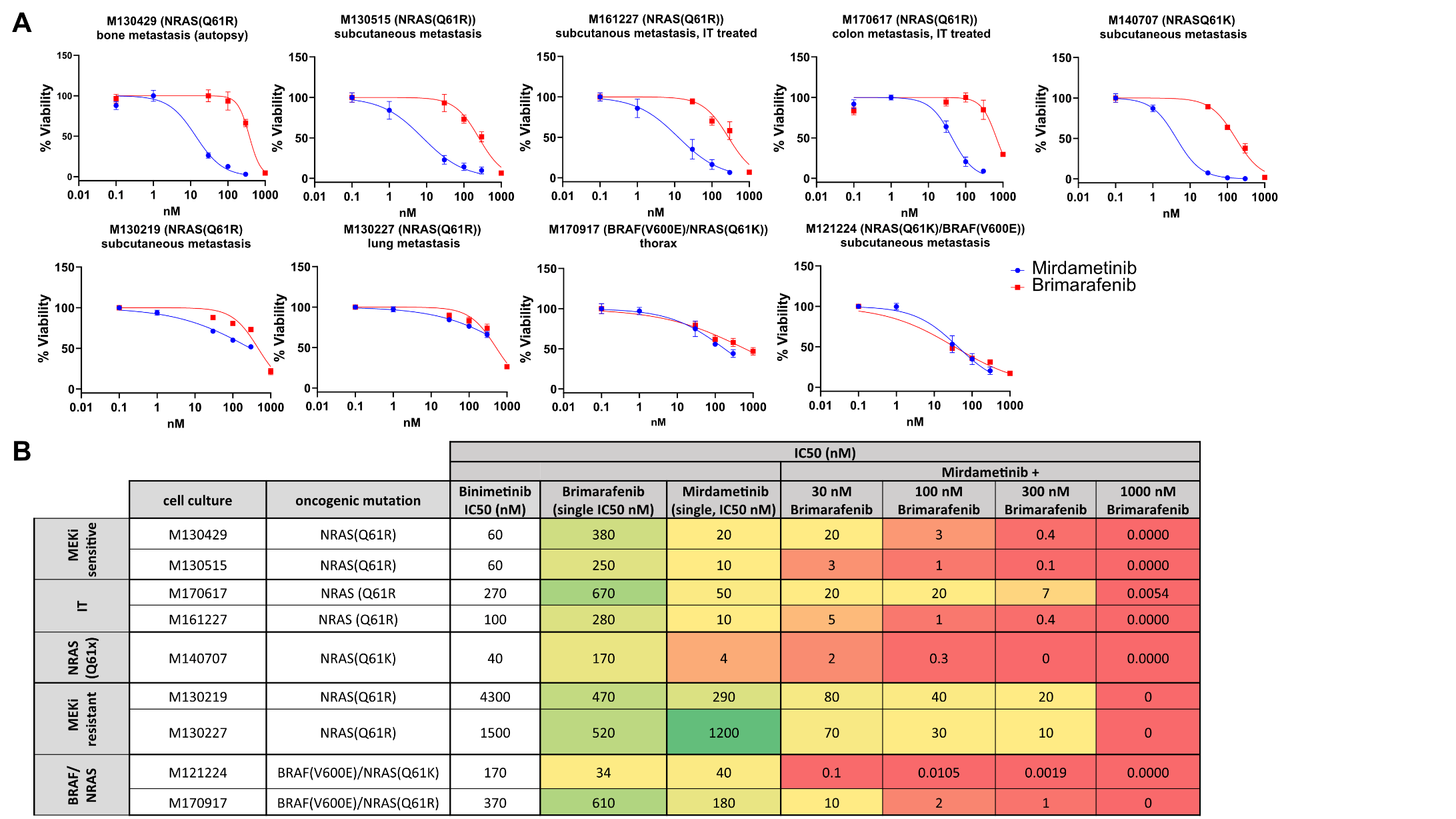


**Supplementary Figure S1: Dose-dependent growth inhibition and IC₅₀ summary for mirdametinib and brimarafenib.** **(A)** Individual growth inhibition curves for patient-derived melanoma cell lines treated with increasing concentrations of mirdametinib or brimarafenib. Data represent the mean of technical triplicates from two independent experiments. Curves and IC₅₀ values were generated using GraphPad Prism software. **(B)** Summary of IC₅₀ values (nM) for binimetinib, mirdametinib, and brimarafenib, either alone or in combination. IC₅₀ values were derived from the dose–response data shown in Figure 1A–E.


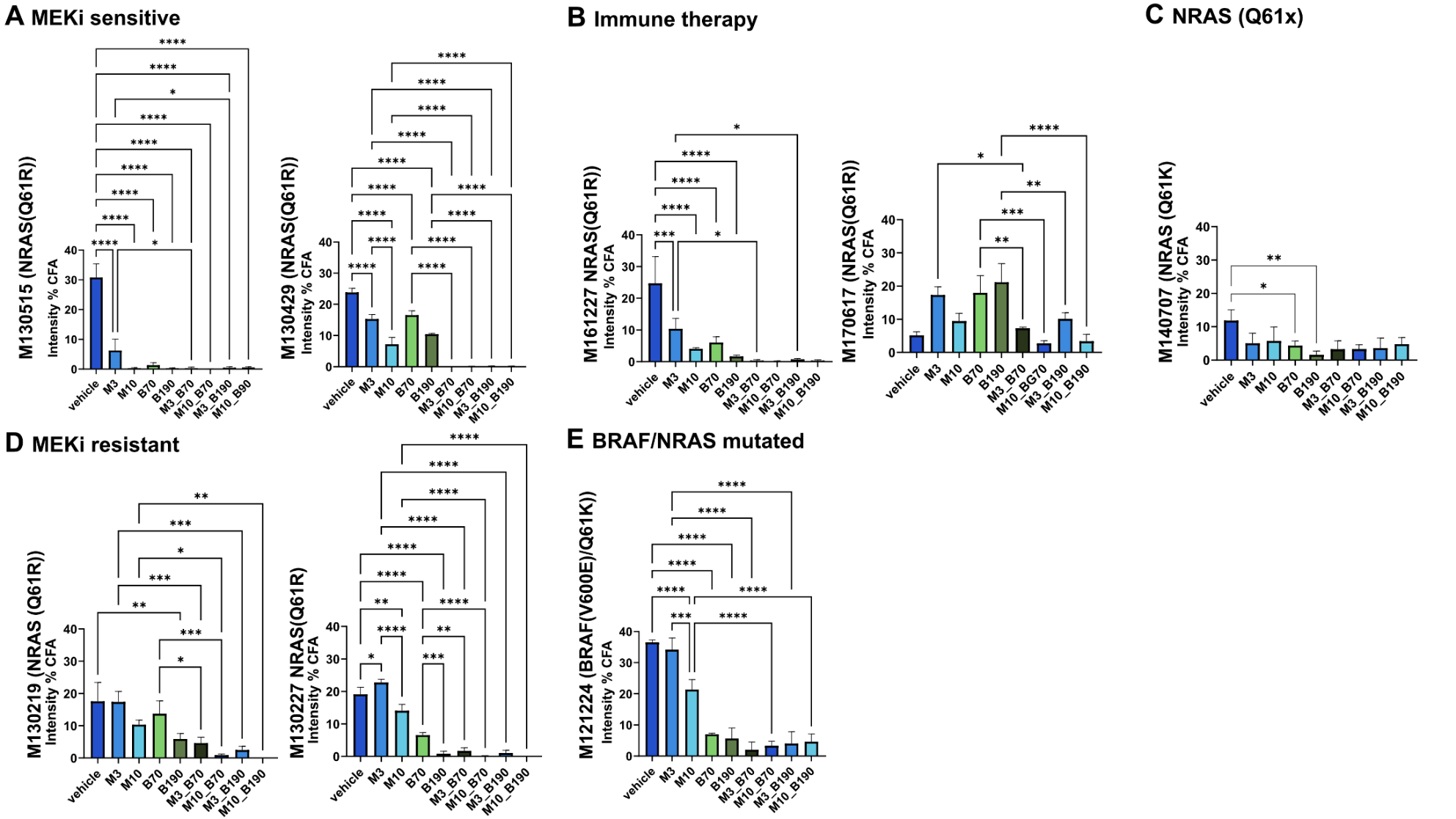


**Supplementary Figure S2:** **Quantification of colony formation following treatment with brimarafenib and mirdametinib**. (A–E) Bar graphs showing the percentage intensity output from the colony formation assay (CFA) corresponding to the experiment presented in Figure 2. Each bar represents the mean of two independent experiments, each performed in technical triplicate.(p < 0.05, *p < 0.01, **p < 0.001, ***p < 0.0001; two-way ANOVA). Vehicle=control wells treated with 0.2% DMSO, M3=mirdametinib (3nM), M10=mirdametinib (10 nM), B70=brimarafenib (70 nM), B190=brimarafenib (190 nM), M3_B70=mirdametinib (3 nM)+binimetinib (70 nM), M10_B70= mirdametinib (10 nM)+binimetinib (70 nM), M3_B190=mirdametinib (3 nM)+binimetinib (190 nM), M10_B190= mirdametinib (10 nM)+binimetinib (190 nM).
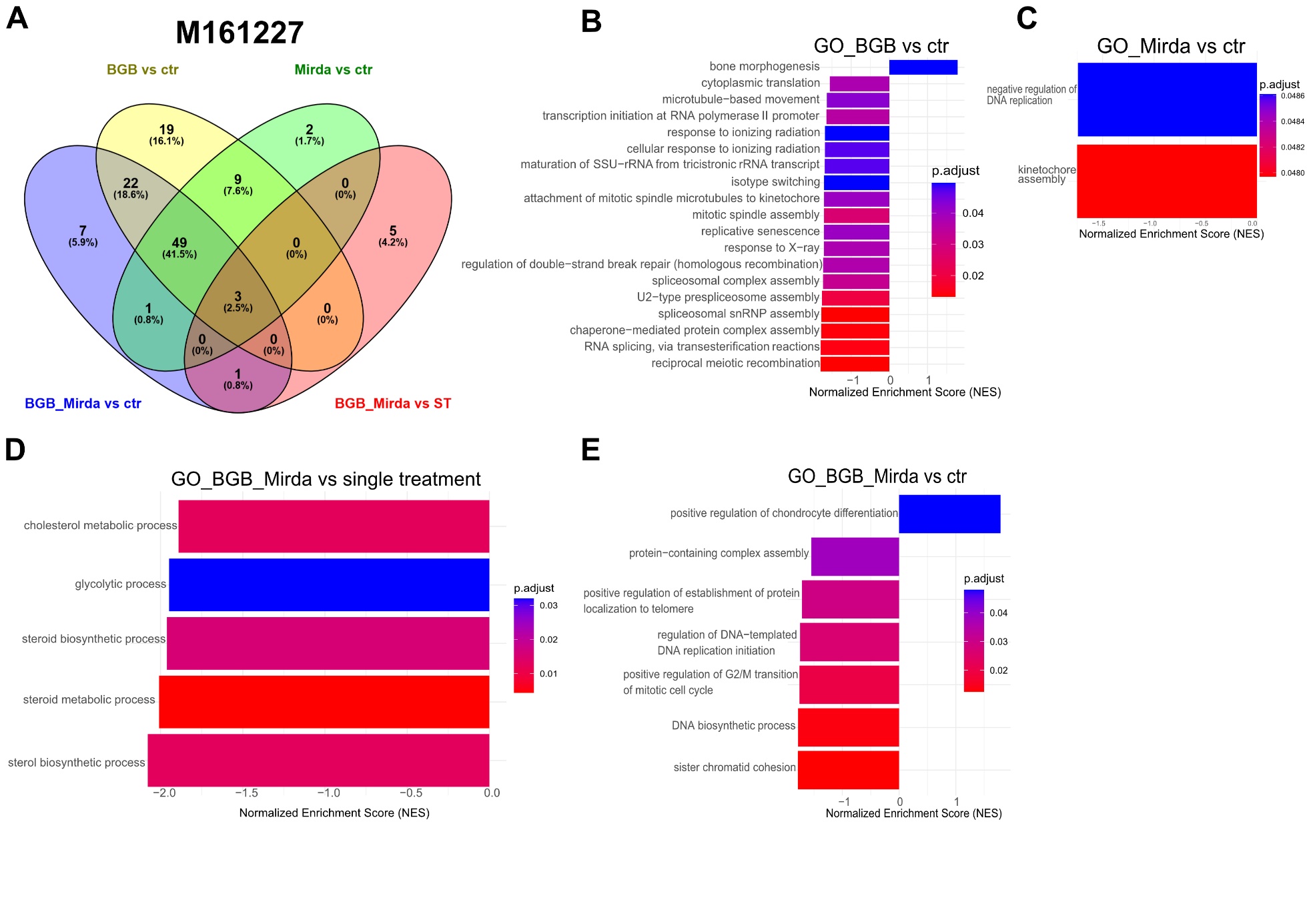


**Supplementary Figure S3: Gene Set Enrichment Analysis of M161227 following treatment with brimarafenib and mirdametinib.** (A) Venn diagram comparing enriched GO Biological Processes (see Supplementary Table S5) in cell line M161227 for the following comparisons: brimarafenib vs. control (BGB vs. ctr), mirdametinib vs. control (Mirda vs. ctr), combination vs. vehicle control (0.2% DMSO) (BGB_Mirda vs. ctr), and combination vs. single treatments (BGB_Mirda vs. ST). (B–E) GO Biological Processes exclusive to each comparison, as identified in the Venn diagram, are shown as bar graphs. Bar height represents the normalized enrichment score (NES), and bars are color-coded according to adjusted p-value. Plots were generated using the R package ggplot2 [25].


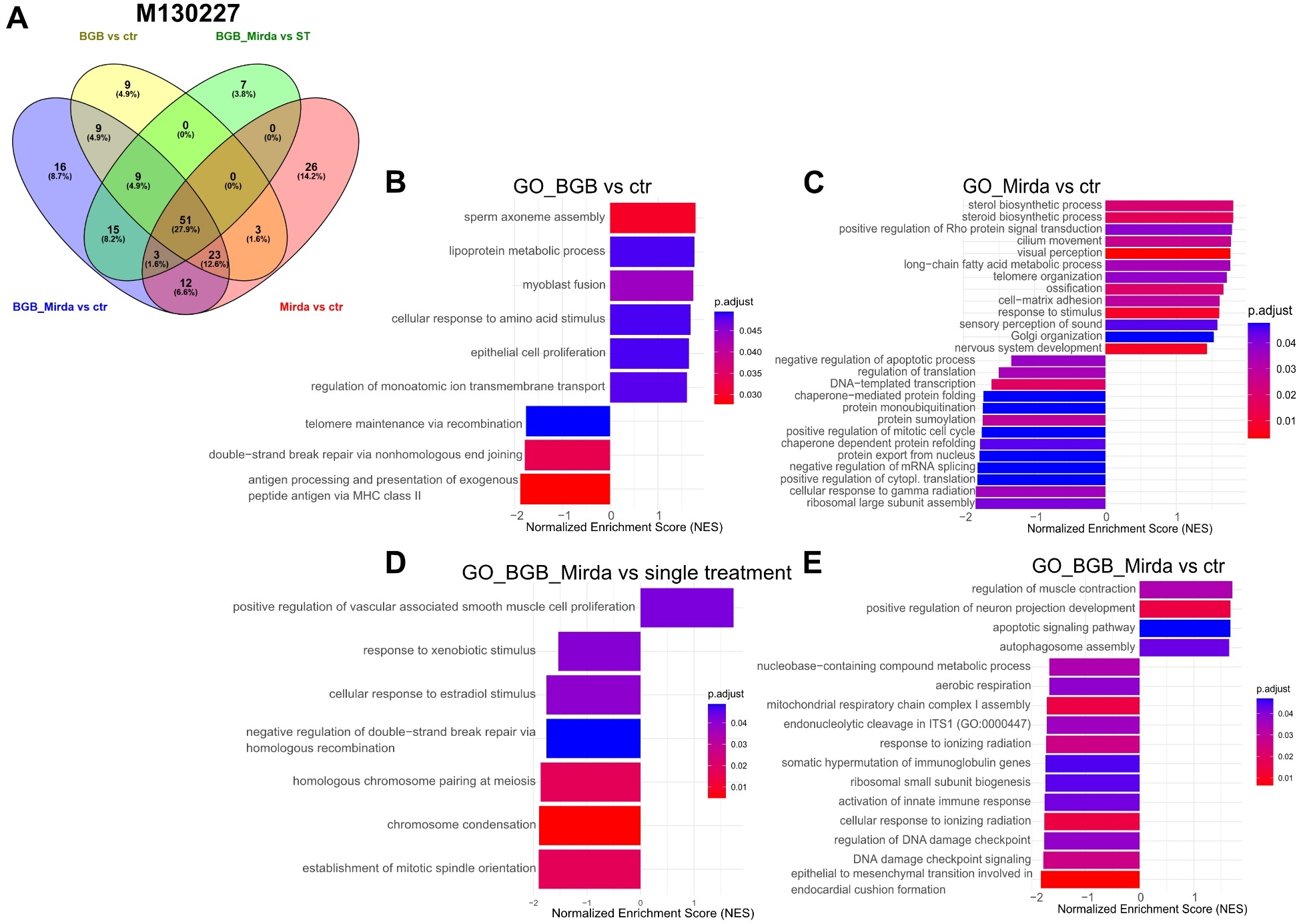


Supplementary Figure S4: **Gene Set Enrichment Analysis of M130227 following treatment with brimarafenib and mirdametinib**. (A) Venn diagram comparing enriched GO Biological Processes (see Supplementary Table S4) in cell line M130227 for the following comparisons: brimarafenib vs. control (BGB vs. ctr), mirdametinib vs. control (Mirda vs. ctr), combination vs. vehicle control (0.2%DMSO) (BGB_Mirda vs. ctr), and combination vs. single treatments (BGB_Mirda vs. ST). (B–E) GO Biological Processes exclusive to each comparison, as identified in the Venn diagram, are shown as bar graphs. Bar height represents the normalized enrichment score (NES), and bars are color-coded according to adjusted p-value. Plots were generated using the R package ggplot2 [25].


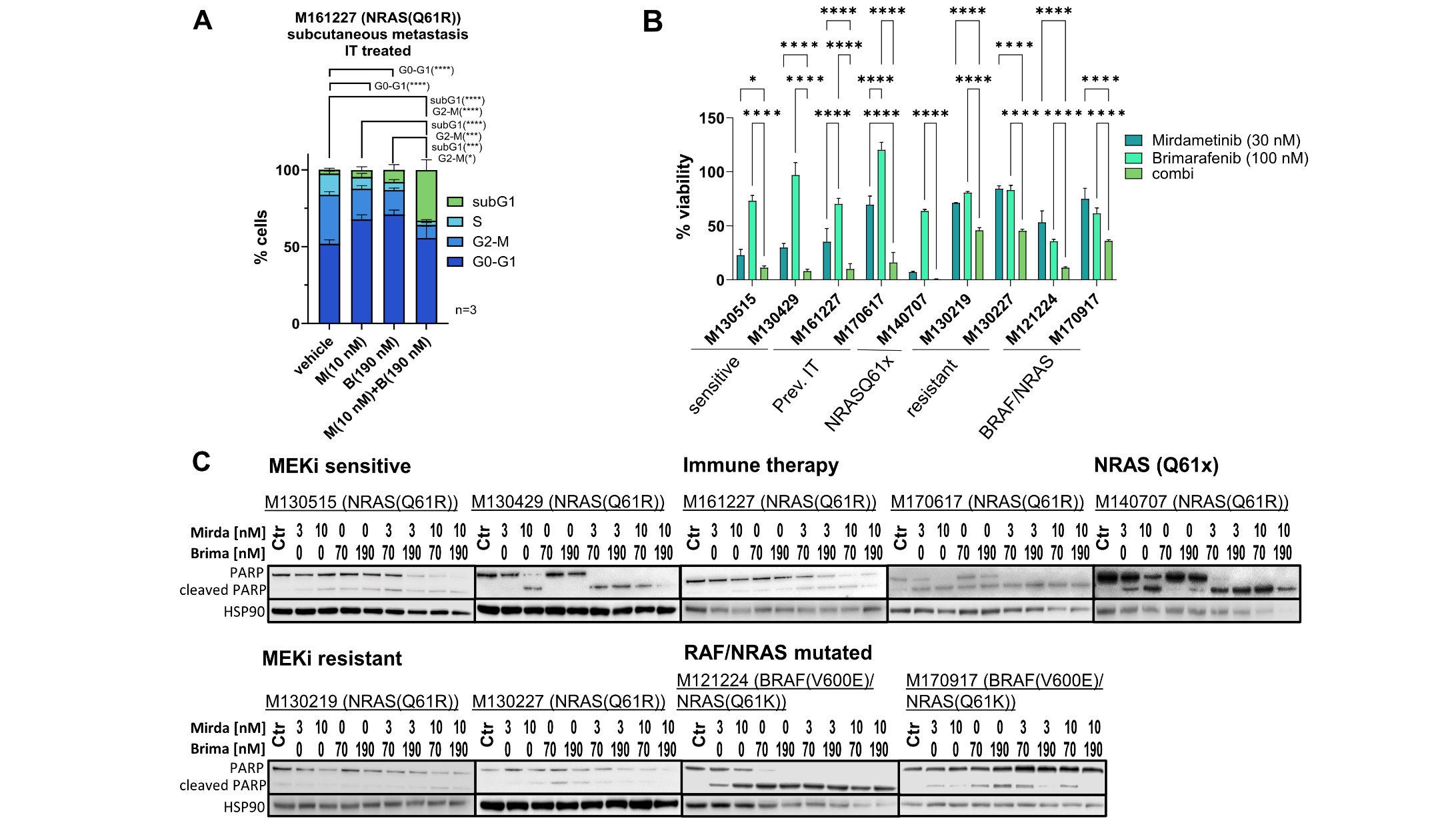


**Suppl. Figure S5**: **Cell cycle analysis and apoptosis induction following treatment with mirdametinib and brimarafenib**. (A) Cell cycle distribution analysis of M161227 assessed by flow cytometry using propidium iodide (PI) staining. Cells were treated for 72h with indicated compounds (M(10nM)=mirdametinib 10 nM; B(90 nM)=brimarafenib 90 nM) or vehicle (0.2% DMSO). Data represent the summary of three independent experiments. Statistics was performed on cell cycle phases indicated (p*<0.05, p***<0.001, p****<0.0001, 2-way ANOVA). ((B) Frequency of viable melanoma cells after treatment with mirdametinib (30 nM), brimarafenib (100 nM) or the combination normalized to vehicle control (0.2% DMSO). (p*<0.05, p**<0.01, p***<0.001, p****<0.0001, 2-way ANOVA). (C) Western blot analysis of PARP cleavage following treatment with various concentrations of mirdametinib, brimarafenib, and their combination.


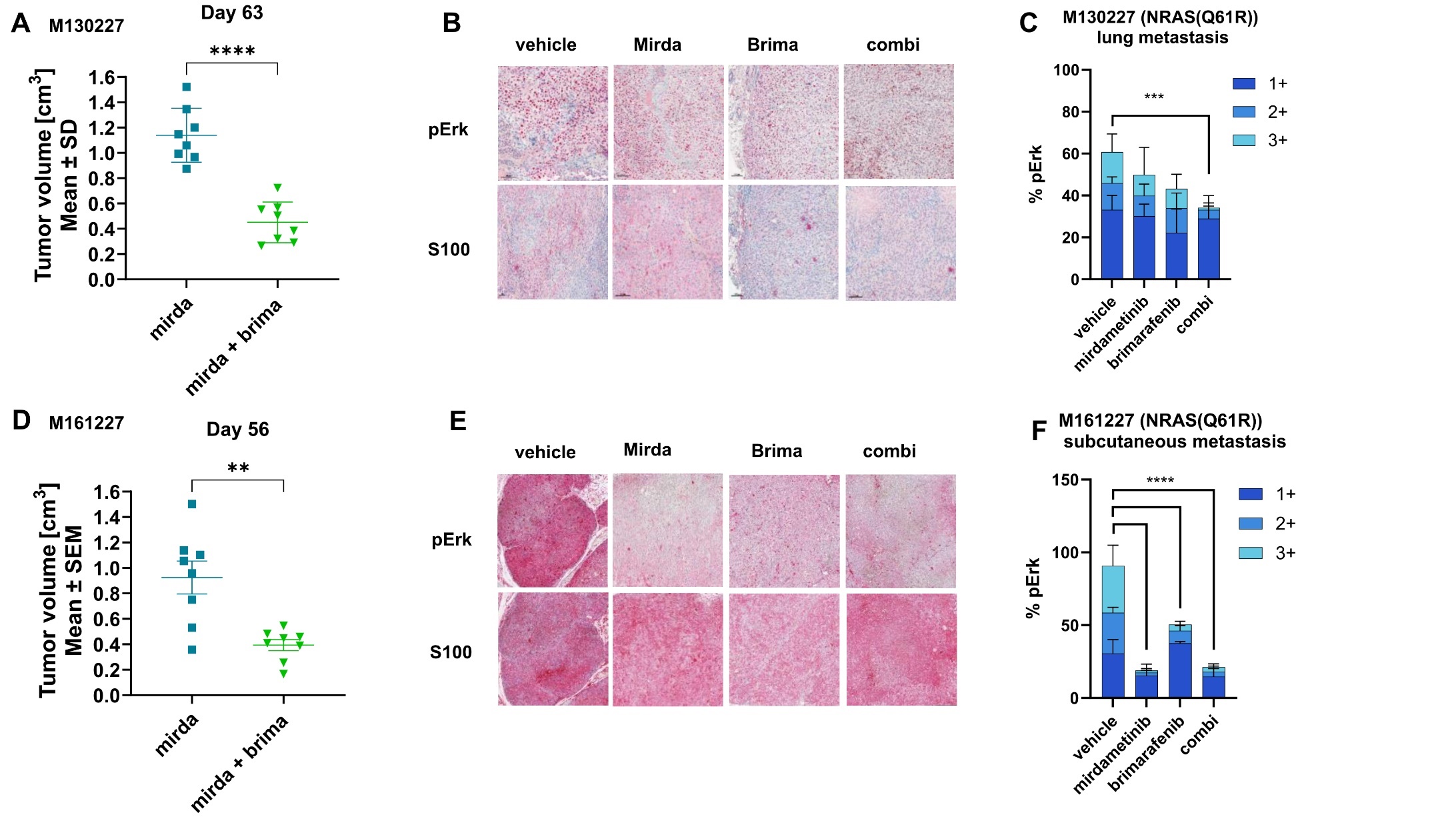


**Suppl. Figure S6: Tumor volume and immunohistochemical analysis of xenograft tumors.** (A,D) Tumor volumes for individual mice on the final day of treatment (day 63 for M130227 and day 56 for M161227) following mirdametinib or combination therapy. (B,E) Representative immunohistochemistry images of pERK and S100 staining in xenograft tumor tissues (B: M130227, E: M161227). (C,F) Quantification of immunohistochemistry staining intensities using QuPath software for M130227 (C) and M161227 (F). Staining intensities were automatically classified using threshold levels of 0.1 = 1+ (weak), 0.2 = 2+ (moderate), and 0.4 = 3+ (strong). Statistics performed on 3+ scoring (p***<0.001, p****<0.0001, 2-way ANOVA).


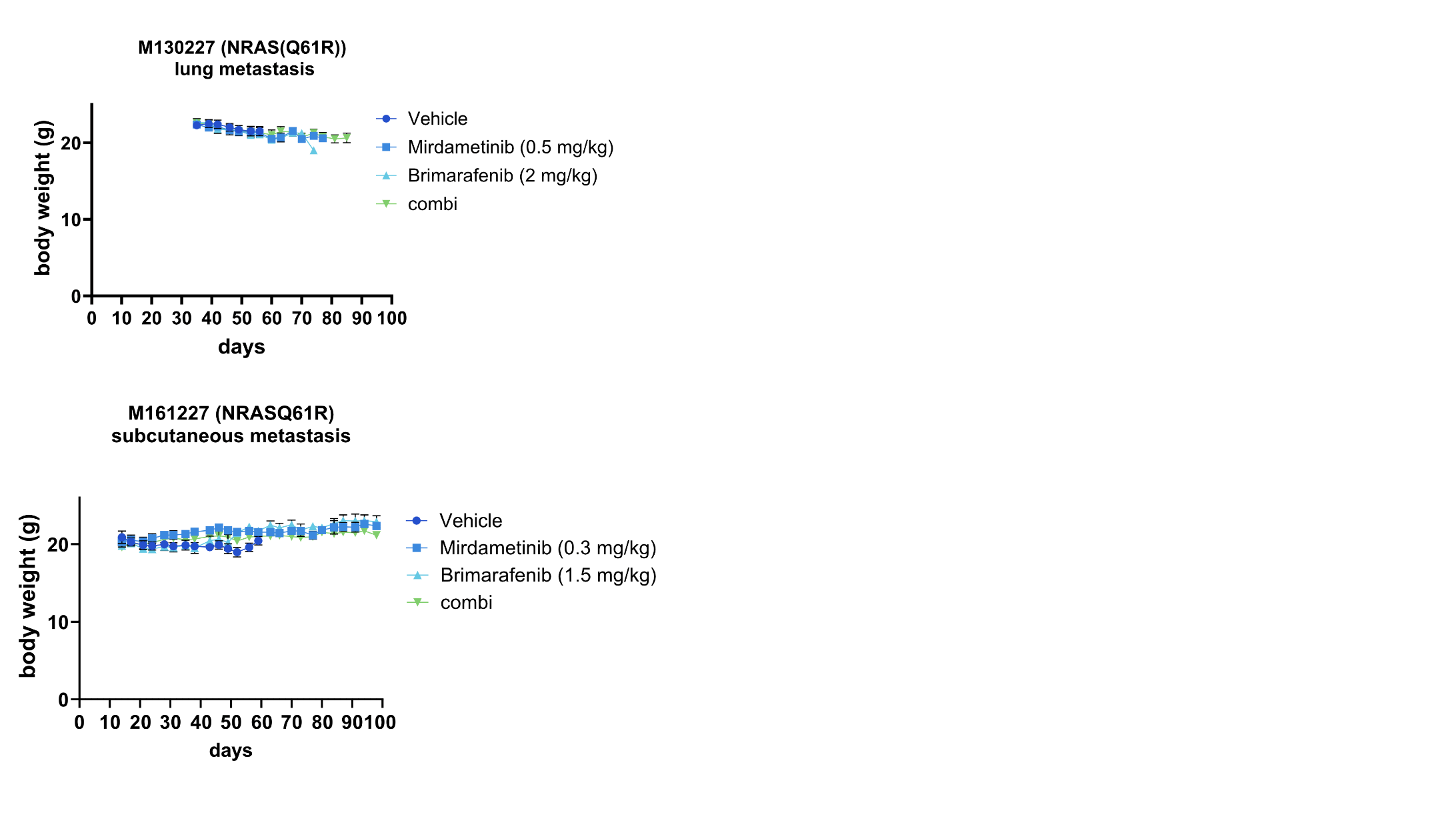


**Suppl. Figure S7**: Body weight of animals measured during the *in vivo* experiments

**Figure legend suppl. Tables**

**Suppl. Table S1:** Differentially expression analysis (DEseq) between Mirda (mirdametinib)_BGB (brimarafenib) combination and vehicle (DMSO), Mirda (mirdametinib) vs DMSO, BGB (brimarafenib) vs DMSO, Mirda_BGB vs DMSO in melanoma cell line M130227

**Suppl. Table S2:** Differentially expression analysis (DEseq) between Mirda (mirdametinib)_BGB (brimarafenib) combination and vehicle (DMSO), Mirda (mirdametinib) vs DMSO, BGB (brimarafenib) vs DMSO, Mirda_BGB vs DMSO in melanoma cell line M161227

**Suppl. Table S3:** Combined differentially expression analysis (DEseq) between Mirda (mirdametinib)_BGB (Brimarafenib) combination and vehicle (DMSO) for both melanoma cell lines (M130227 and M161227)

**Suppl. Table S4**: Gene set enrichment analysis for GO_Biological Processes using RNAseq data (DEseq) from cell line M130227

**Suppl. Table S5**: Gene set enrichment analysis for GO_Biological Processes using RNAseq data (DEseq) from cell line M161227

**Suppl. Table S6:** Statistical analysis of data from the *in vivo* Xenograft experiment performed with patient-derived cell line M130227

**Suppl. Table S7:** Statistical analysis of data from the *in vivo* Xenograft experiment performed with patient-derived cell line M161227
